# First Insight into the Natural Attenuation of Emerging Contaminants Using a Metagenomics Approach from Drinking Water Sources in the Free State

**DOI:** 10.3390/microorganisms13102349

**Published:** 2025-10-14

**Authors:** Avela Mqambalala, Maleke Maleke, Lore-Mari Deysel, Jorge R. Osman, Alba Gomez-Arias, Angel Valverde, Julio Castillo Hernandez

**Affiliations:** 1 Department of Microbiology and Biochemistry, University of the Free State, Bloemfontein 9300, South Africa; avelamqambalala@gmail.com; 2 Department of Life Sciences, Central University of Technology, Free State, Bloemfontein 9300, South Africa; mmaleke@cut.ac.za; 3 Institute of Groundwater Studies, University of the Free State, Bloemfontein 9300, South Africa; cruywagenlm@ufs.ac.za; 4 Instituto de Geología Económica Aplicada, Universidad de Concepción, Concepción 4070386, Chile; osman.jorge@gmail.com; 5 Instituto de Recursos Naturales y Agrobiología de Sevilla (IRNAS-CSIC), 41012 Sevilla, Spain; albita.anortita@gmail.com; 6 Instituto de Recursos Naturales y Agrobiologıa de Salamanca (IRNASA-CSIC), 37008 Salamanca, Spain; avalverdeportal@gmail.com; 7 Department of Integrated Science, University of Huelva, 21007 Huelva, Spain

**Keywords:** emerging contaminant, genes, natural attenuation, biodegradation, indigenous microbes

## Abstract

Emerging contaminants have gained interest over the years due to their adverse effects on the aquatic environment. Therefore, it is essential to improve the current strategies for their removal. Biodegradation has emerged as an efficient strategy driven by microorganisms through metabolism and co-metabolism pathways. Enzymes encoded by specific genes facilitate these processes. This study aimed to identify and quantify the genes involved in these pathways. The research identified bacterial species belonging to the genera *Pseudomonas*, *Nitrosomonas*, *Nitrosospira*, and *Methylotenera*, which are associated with the degradation of emerging contaminants. Additionally, it successfully identified genes linked to metabolism and co-metabolism processes within the indigenous bacteria (MAGs). The findings suggest that the native bacteria in the samples may have the natural potential to mitigate emerging contaminants in aquatic environments through the combined actions of metabolism and co-metabolism.

## 1. Introduction

Emerging contaminants (ECs) have gained attention over the years due to their negative impact on aquatic and terrestrial life [1]. ECs have been seen to negatively affect gill and kidney cells in freshwater fish and trout, and in humans they have been associated with inhibiting the growth of embryonic kidney cells [2]. These ECs consist of various organic compounds derived from pharmaceuticals, personal care products, pesticides, and endocrine-disrupting compounds that enter aquatic ecosystems daily due to intense human activity [1,2,3]. South Africa mainly relies on surface water, with groundwater contributing only 15% of the country’s total water usage [4]. In the Free State province, the Rustfontein and Welbedacht dams are part of the Modder River catchment and serve as critical water sources for domestic, agricultural, and industrial needs in Bloemfontein and nearby townships. Agricultural and industrial activities and the absence of a sewage system in the townships contaminate these water sources with ECs [5,6]. For instance, ECs such as pesticides (atrazine (1237 ng/L) and terbuthylazine (1969 ng/L)), pharmaceutical products (atenolol (25,900 ng/L) and sulfamethoxazole (34.5 µg/L)), and anti-retrovirals (nevirapine (1480 ng/L), zidovudine (973 ng/L), lamivudine (242 ng/L), and stavudine (778 ng/L)) have been detected in surface, ground, and wastewater from South Africa in the Free State, Gauteng, and North West provinces [7,8,9].

These contaminants can have a detrimental impact on the microbiome in aquatic ecosystems [1,10]. Emerging contaminants seem to shift microbial diversity, increasing richness but decreasing diversity, and have been associated with alterations in microbial respiration rate and gene expression [11,12,13,14]. For instance, anaerobic respirations such as methanogenesis, sulphate, and nitrogen reduction can be affected by antibiotics [15]. Keen and Patrick (2013) reported that the presence of antibiotics could either inhibit or stimulate the activity of sulphate reducers, depending on the concentration, thus influencing the overall sulphate reduction process in contaminated environments [16]. Additionally, ECs like naphthalene and phenanthrene triggered the upregulation of antimicrobial resistance genes, including class I integrons (*intI1*), sulphonamide resistance genes (*sul1*), and aminoglycoside resistance genes (*aadA2*). Bacteria seem to utilize this mechanism to survive in the presence of these pollutants [12,13].

Bacterial species also possess specific genes that encode enzymes capable of biodegrading ECs, which can be described as when these emerging contaminants are broken down into simple molecules such as carbon dioxide, which helps reduce their toxicity and, in some cases, improve water quality. Various genera, including *Pseudomonas*, *Bacillus*, *Nitrosomonas*, *Nitrospira*, *Methylomirabilis*, and *Methylosinus*, have demonstrated the ability to biotransform ECs through reductive and oxidative metabolism pathways. These pathways seem to be regulated by the seasonal variation (particularly dry and wet seasons), which influences temperature, rain season, oxygen availability, nutrient levels, EC concentrations, and the types of microorganisms present in the aquatic environment, all of which may promote or hinder the effectiveness of EC biodegradation. For instance, Matamoros and Rodríguez (2017) reported that the in-stream natural attenuation of ECs showed a high seasonality with half-lives of 5.1 ± 4.0 h for the cold season and 2.7 ± 2.3 h for the warm season [17]. Moreover, Wooding et al. (2017) [18] highlighted that precipitation during the wet season in South Africa leads to increased water levels in rivers, which can enhance the transport and dilution of contaminants. This dilution effect might facilitate the bioavailability of ECs, making them more accessible to planktonic microorganisms for degradation [18].

Influences of seasonal variation on oxygen availability indicate whether aerobic or anaerobic conditions will prevail. This, in turn, influences the microbial diversity and affects the biodegradation of ECs. Oxidative metabolism is considered more favorable for the degradation of many ECs. However, certain aerobically recalcitrant contaminants could still be biodegraded under anaerobic conditions, indicating that anaerobic processes can be effective under specific circumstances [19]. It is worth mentioning that anaerobic processes often result in a more complex array of metabolites (some still toxic) than aerobic biodegradation, which tends to produce fewer intermediate compounds. This pathway is driven by two mechanisms, which are the hydrolytic dechlorination pathway, which is similar to the aerobic pathway, and the reductive dechlorination pathway. These pathways produce six metabolites, including hydroxy atrazine, deethylatrazine, *N*-isopropylammelide, deisopropylatrazine, cyanuric acid, and the novel metabolite 4-ethylamino-6-isopropylamino-1,3,5-triazine (EIPAT), which were detected in the anaerobic metabolism pathways of atrazine. In contrast, three intermediate compounds were detected in the aerobic metabolism pathways [20,21]. Under aerobic conditions, the hydrolytic dechlorination is catalyzed by atrazine chlorohydrolase (*atzA* or *trzN*) to produce hydroxyatrazine (HA). Hydroxyatrazine is converted into two different aminohydrolases encoded by *atzB* and *atzC*. After that, cyanuric acid is metabolized into CO_2_ by the hydrolytic reactions encoded by *trzD*/*atzD*, *trzE*/*atzE*, and *trzF*/*atzF* [22,23].

The nutrient bioavailability and EC concentrations are also influenced by seasonality. The biotransformation or degradation of some nutrients (e.g., ammonium or sulphur) or contaminants (e.g., toluene) by microbial cultures can induce co-metabolism pathways where enzymes involved in the metabolism of both substrates also fortuitously convert ECs as secondary metabolites, which results in their partial or complete degradation. For example, the bioavailability of ammonia can facilitate the co-metabolism degradation of sulfamethoxazole through the nitrification process facilitated by different bacteria such as *Rhodococcus rhodochorus*, *Pseudomonas aeruginosa*, and *Rhodococcus equi* [24,25]. This process is mainly driven by ammonia monooxygenase (*amoA*), which can co-metabolise this compound with ammonia [24,26].

Seasonal changes associated with biological and chemical factors appear to play a crucial role in regulating metabolism and co-metabolism, contributing to the biodegradation of ECs. However, no studies have yet demonstrated the impact of this variation on EC biodegradation in natural aquatic environments. It is well known that microbial communities capable of degrading organic contaminants may serve as natural barriers in breaking down ECs [27]. Biomarker genes have been used to predict such biodegradation. For instance, Gedalanga et al. 2014 targeted the multicomponent monooxygenase gene in *Pseudonocardia dioxanivroans* CB1190, which was used as a biomarker to identify the potential for 1,4-Dioxane biodegradation [28]. This study highlights the use of genes as biomarkers of potential EC biodegradation. This study employed shotgun metagenomics analysis to examine the shifts in genes involved in ECs biodegradation during the dry and wet seasons in the Free State, South Africa Rivers. We hypothesize that seasonal variation may influence the abundance and activity of genes related to EC biodegradation, mediated by metabolism and co-metabolism pathways. We suggest that the variation might also promote co-metabolism as the more efficient pathway for EC microbial biodegradation. Additionally, it proposes that bacterial communities may act as natural barriers to EC contamination, regardless of seasonal changes.

## 2. Materials and Methods

### 2.1. Site Description

The Rustfontein Dam (E 26°37′11″ S 26°16′15″) is a gravity-type dam, and the Welbedacht Dam (E 26°37′11″ S 29°54′34″) is a concrete-type dam located at the Modder River catchment. The ECs detected included carbamazepine, atrazine, metolachlor, terbuthylazine, and 17-alpha-ethinyl-estradiol (Table 1) [5,6]. The selected ECs for this study were highly frequent during summer and winter in the dams, as shown in the survey by Oke and co-workers. In this region, summer temperatures range from 12 °C to a maximum of 30 °C. The average minimum temperature in winter is 3 °C, while the maximum reaches 18 °C. Rainfall occurs mainly in the summer, with winters being dry. The vegetation in this province is mostly grassland [29].

### 2.2. Water Sample Collection

Due to the EC concentrations found in the dams of the Modder River catchment, the Rustfontein and Welbedacht dams were selected for this study. The raw surface water samples were collected at the abstraction point using sterile 5 L plastic bottles in triplicate from each dam in the wet and dry seasons (total n° of samples 12). Previously, the 5 L plastic bottles were rinsed three times with raw water in the water collection. The samples were then transported to the Department of Microbiology and Biochemistry and stored at 4 °C. The samples were used downstream for physico-chemical characterization and microbial analysis.

### 2.3. Physico-Chemical Characterisation

Physicochemical parameters such as pH, Electrical Conductivity (EC), Total Dissolved Solids (TDS), redox potential (Eh), and temperature (°C) were measured on-site to avoid the oxidation process, using an ExStix^®^II multi-probe (Extech Instruments, Nashua, NH, USA) and an ExStix^®^II Eh probe (Extech Instruments, USA). Eh, measurements were corrected accordingly to Standard Hydrogen Electrode (SHE). The Dissolved Oxygen was measured using the Aquaread AP-2000 multiparameter probe (Aquaread Ltd., Broadstairs, UK). The DOC (Dissolved Organic Carbon) was analyzed using the fully automated multi-N/C UV HS system (analytikjena^TM^, Jena, Germany). The samples were loaded via flow injection using parallel plunging. The DOC was measured at 254 and 185 nm. Energy sources such as SO_2_^4−^ concentrations were analyzed on-site using a HACH spectrophotometer (model DR/900 colorimeter) (Johannesburg, South Africa) according to the turbidimetric methods described in the HACH Procedures Manual (Method Sulphate 608). The NO_3_ and NH_4_ concentrations were measured using the Systea Easychem 200–Discreet analyzer (automated spectrophotometer) (Systea^TM^, Anagni, Italy). The NO_3_ and NH_4_ concentrations were analyzed by the Institute of Ground Water Studies (IGS) at the University of the Free State.

### 2.4. Environmental DNA Extraction and Quality Assessment

Raw water samples (1 L) were filtered using a vacuum system through a sterile 0.2 µm cellulose nitrate filter (Sartorius Stedim Biotech GmbH, Göttingen, Germany) to concentrate the cells. The filters were stored in Petri dishes at −22 °C until DNA extraction was conducted. The rest of the water samples were stored at 4 °C. The stored filters were cut into pieces using a sterile surgical blade under aseptic conditions. Genomic DNA was extracted using a combined CTAB and TAN Bead Maelstrom 8 (M8-H) method. A small portion (1 μL) of the purified eDNA was quantified using the Qubit^TM^ 2 Fluorometer (Invitrogen, Life Technologies, Waltham, MA, USA) with the Qubit^®^ 1x dsDNA HS Assay Kit (Invitrogen, Life Technologies) (see Table S1 for concentration values). The eDNA integrity was visualized using gel electrophoresis. The remaining eDNA was shipped to Mr. DNA (info@mrdnalab.com, USA) for sequencing.

### 2.5. Metagenomic Shotgun Sequencing

The initial concentration of eDNA was quantified using the Qubit^®^ dsDNA HS Assay Kit (Life Technologies, Carlsbad, CA, USA). Due to the low eDNA concentration, an amplification was carried out using the Repli-g Midi Kit (Qiagen, Hilden, Germany) and followed by purification of the amplified reaction using the DNeasy PowerClean Pro Cleanup Kit (Qiagen). The amplified and cleaned DNA concentration was quantified using the Qubit^®^ dsDNA HS Assay Kit (Life Technologies). 50 ng of DNA was used to prepare the library using the Illumina DNA Prep (M) tagmentation library preparation kit (Illumina, San Diego, CA, USA) following the manufacturer’s user guide. The samples underwent the simultaneous fragmentation and addition of adapter sequences. These adapters are utilized during a limited-cycle PCR in which unique indices are added to the sample. Following the library preparation, the final concentration of the libraries (Table S1) was measured using the Qubit^®^ dsDNA HS Assay Kit (Life Technologies), and the average library size was determined using the Agilent 2100 Bioanalyzer (Agilent Technologies, Santa Clara, CA, USA). The libraries were then pooled in equimolar ratios of 0.6 nM and sequenced paired-end for 500 cycles using the NovaSeq 6000 system (Illumina) (info@mrdnalab.com, USA).

### 2.6. Bioinformatic Analysis

#### 2.6.1. Quality Check, Filtering, Assembling, and DiTing

The quality of the sequenced reads was assessed with FastQC v0.11.6, and low-quality bases (per base sequence quality > 90) were removed with Trimmomatic v0.36 [30,31]. The filtered reads were merged using PEAR with a minimum specified overlap of 10 bases, a minimum assembled length of 50 bases, and a minimum alignment *p*-value of 0.01, resulting in an average assembly efficiency of 99% [32]. During this merging, the sequencing depth was normalized across samples. The contigs were assembled using metaSPAdes v3.11.0 [33]. The contig quality was assessed using MetaQUAST [34].

The contigs were analysed using the software DiTing v0.9 to infer and compare biogeochemical pathways [35]. DiTing annotated protein sequences based on the KEGG Orthology database for most microbial-mediated biogeochemical cycles (e.g., carbon, nitrogen, sulphur, and metals) [36]. Genes were predicted and translated from the assembled contigs by Prodigal v2.6.3 with the “-p meta” option [37]. The relative abundance of each functional gene was calculated, followed by the relative abundance of each pathway, which was calculated according to a customized formula [35]. Heat maps and sketch plots were also generated with DiTing for easier visualisation and comparison of the biogeochemical cycles.

#### 2.6.2. Taxonomic Assignment and Identification of Biodegradation Pathways

The merged sequences were aligned to long reads using bowtie2 [38,39]. The taxonomy assignment (classification) and relative abundance were determined using MetaPhlan, which profiles the bacterial composition from the metagenomic data, specifically up to the species level [40,41]. It is important to note that this study will focus exclusively on the bacterial kingdom. MetaPhlan was accessed using Humann3, a pipeline utilized to determine the presence or absence of microbial pathways in a community from metagenomics data [41]. The ChocoPhlan and Uniref90 databases were used to predict the presence of the xenobiotic biodegradation pathway [41]. The recommended parameters provided by the developers were applied to all tools used in the analysis. The relative abundance of the taxonomic assignment and pathways was then visualized using R-studio.

#### 2.6.3. Binning, MAGs, and Functional Annotations

Contigs were organised into bins, or the metagenome-assembled genomes (MAGs), based on tetra-nucleotide sequence composition with MetaBAT2 v2.11.3 [42]. The completeness, coverage, contamination, and strain heterogeneity of the MAGs were estimated using CheckM v1.0.11 with the single-copy marker gene [30]. The MAGs were the selected bins with a completeness greater than 75% and contamination less than 5% that were selected for further analysis. To check the presence or absence of the genes associated with emerging contaminant degradation, the selected MAGs were deposited in RAST. Then, BLAST v2.15.0 aligned the genome bins to reference sequences of characterized genes of interest. The sequences of genes of interest were obtained from Uniprot (https://www.uniprot.org/).

## 3. Results

### 3.1. Physico-Chemical Characterization

The water from the Rustfontein and Welbedacht dams exhibits oxidative (Eh ranging from 160 to 200 mV and DO ranging from 4 to 12 mg/L) and circumneutral to slightly alkaline (pH between 7.2 and 7.8) conditions (Table 2). The electrical conductivity was significantly higher in the Welbedacht Dam (41.51 mS/m) compared to the Rustfontein Dam (17.13 mS/m), which may be due to anthropogenic activity in the dam. However, the seasonal variation in all these parameters was insignificant. Contrary to the pattern of other parameters, DOC concentrations were higher in winter in both the Rustfontein and Welbedacht dams (2.8 mg/L and 4.2 mg/L, respectively) compared to summer (2.71 mg/L and 3.62 mg/L). Additionally, DOC concentrations were significantly higher in the Welbedacht Dam than in the Rustfontein Dam. Sulphate concentrations followed the same pattern, while nitrate showed an opposite trend (Table 2). Similar to most parameters, seasonal variations were insignificant. Interestingly, ammonium (NH_4_) concentrations followed their pattern, higher in summer and negligible in winter in both dams (Table 2).

### 3.2. Bacterial Diversity

**Figure 1:**
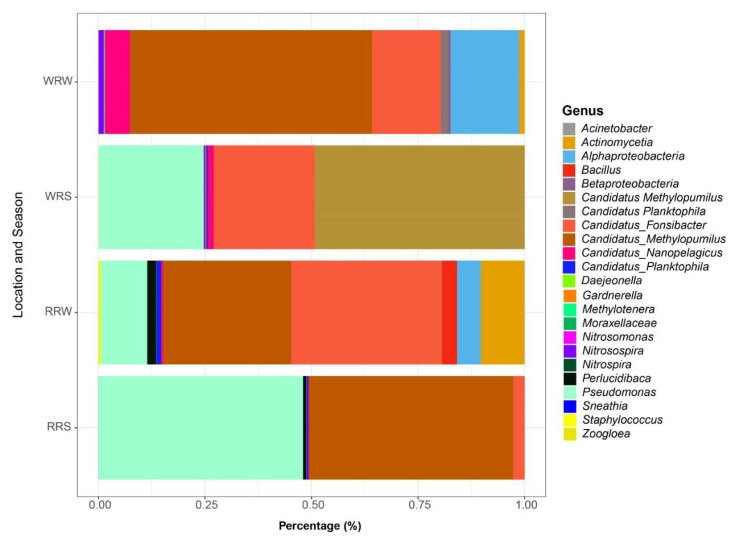
The microbial diversity plot of the Rustfontein and Welbedacht dam samples for summer and winter. RRS represents Rustfontein Raw Summer, RRW represents Rustfontein Raw Winter, WRS represents Welbedacht Raw Summer, and WRW represents Welbedacht Raw Winter. The plot was generated from data obtained from MetaPhlan (Supplementary Tables S2–S5).

Rustfontein and Welbedacht dams appear to be predominantly colonized by the phylum Proteobacteria (46%), with lower percentages (<11%) of other phyla such as Planctomycetes, Actinobacteria, Bacteroidetes, and Firmicutes (Figure 1). In the Rustfontein dam, the predominant species (>1%) during the wet season were *Pseudomonas* and *Candidatus methylopumilus*, with an average relative abundance of 34.52%, followed by *Candidatus fonisbacter* (1.89%). The most abundant species during the dry season included *Candidatus fonisbacter* (23.00%), followed by *Candidatus methylopumilus* (19.55%), *Pseudomonas* (7.09%), *Actinomycetia* (6.58%), *Bacillus* (2.29%), and *Perlucidibaca* (1.36%).

Welbedacht Dam seems to share certain species with Rustfontein Dam (Figure 1). In the wet season, the most abundant species was *Candidatus methylopumilus,* with an average relative abundance of 20.01%, followed by *Pseudomonas* (19.95%), *Candidatus fonisbacter* (19.24%), and *Candidatus nanopelagicus* (1.11%). In the dry season, *Candidatus methylopumilus* persists as the more predominant species in this aquatic system, with an average relative abundance of 46.16%. *Candidatus fonisbacter* (13.08%), *Candidatus nanopelagicus* (4.76%), *Candidatus planktophila* (1.42%), and *Actinomycetia* (1.01%). *Pseudomonas* (0.01%) seems to be the least abundant during this season. Although some species show a decrease in abundance across seasons, the overall composition appears to remain unaffected.

### 3.3. Main Biogeochemical Pathways Associated with the Co-Metabolism of ECs

**Figure 2:**
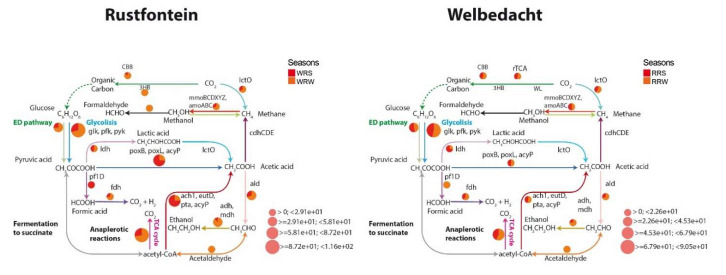
The Diting plots illustrate the presence and relative abundance of carbon cycle-related genes across both summer and winter seasons in the Rustfontein and Welbedacht dams. RRS represents Rustfontein Raw Summer, RRW represents Rustfontein Raw Winter, WRS represents Welbedacht Raw Summer, and WRW represents Welbedacht Raw Winter.

Genes related to critical biogeochemical cycles were identified in all four metagenomes (Figure 2). Interestingly, the relative abundance of the pathways was 27% (Rustfontein dam) and 55% (Welbedacht dam) higher in winter than in summer. As this article focuses on investigating the metabolism and/or co-metabolism associated with the degradation of ECs, only genes and pathways (directly and indirectly) of particular interest are described. Genes and pathways related to carbon cycling were the most abundant in the four metagenomes (Figure 2). Methane metabolism was not well represented in the metagenomes (relative abundance of the enzymes was, on average, <1.4%). Enzymes encoded by genes linked to methanogenesis and methane oxidation, such as acetate kinase (*ackA*) and methane/ammonia monooxygenase (*smmoABC*-*amoABC*), were annotated.

**Figure 3:**
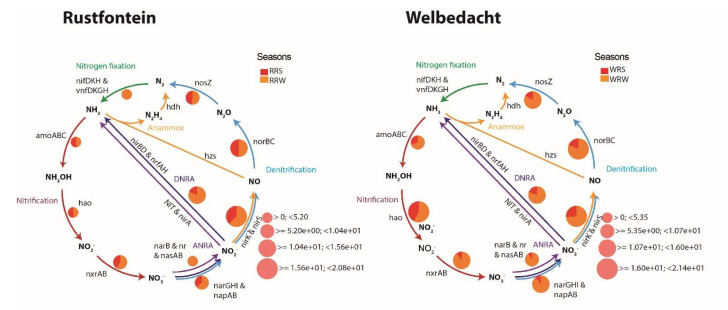
The Diting plots illustrate the presence and relative abundance of nitrogen cycle-related genes across both summer and winter seasons in the Rustfontein and Welbedacht dams. RRS represents Rustfontein Raw Summer, RRW represents Rustfontein Raw Winter, WRS represents Welbedacht Raw Summer, and WRW represents Welbedacht Raw Winter.

Nitrogen metabolism is represented by the dissimilatory nitrate reduction and nitrification pathways, with the latter involving several processes (Figure 3). These include the conversion of ammonia to hydroxylamine, ammonia monooxygenase encoded by *amoABC* gene; the conversion of hydroxylamine to nitrite, catalyzed by hydroxylamine oxidoreductase encoded by *hao* and the conversion of nitrite to nitrate, catalyzed by nitrite oxidoreductase encoded by *nxrAB*. Notably, the complete set of genes (see Figure 3) for full nitrification and complete denitrification was detected in all four metagenomes, regardless of the season. The relative abundance of genes linked to dissimilatory nitrate reduction was higher in winter (3.8%) than in summer (2.5%). In contrast, the abundance of genes associated with the nitrification process showed no seasonal variation.

**Figure 4:**
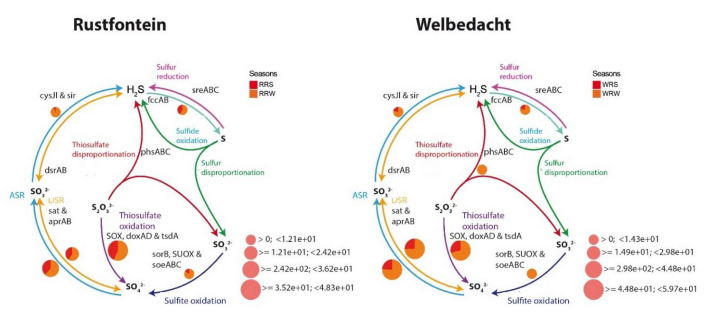
The Diting plots illustrate sulphur cycle-related genes’ presence and relative abundance across both summer and winter seasons in the Rustfontein and Welbedacht dams. RRS represents Rustfontein Raw Summer, RRW represents Rustfontein Raw Winter, WRS represents Welbedacht Raw Summer, and WRW represents Welbedacht Raw Winter.

Due to the oxidative conditions in the dams, the sulphur cycle was mainly driven by the assimilatory sulphate reduction (e.g., *sat*, *aprAB*, *cysJI*, *sir* genes) and sulphur or thiosulphate oxidation (e.g., *fccAB*, *sorB*, *SUOX*, *soeABC* genes) pathways (Figure 4). These pathways represent 2.3% and 5.7% of the functional annotations in the metagenomes, respectively. The sulphide oxidation seems to be coupled with the partial dissimilatory sulphate reduction mediated by the sat and aprAB enzymes. Winter seems to promote sulphur or thiosulphate oxidation and partial dissimilatory sulphate reduction.

### 3.4. Presence of Xenobiotic Degradation Pathways

**Figure 5:**
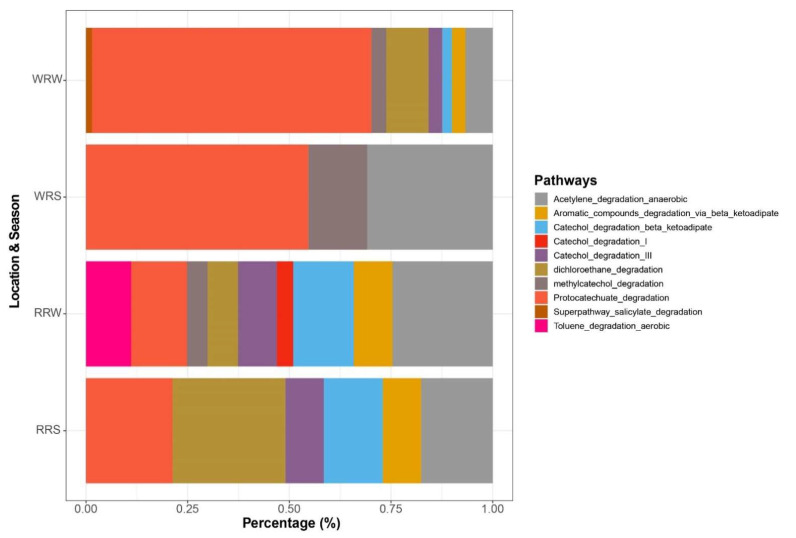
A plot showing the presence of Xenobiotic degradation pathways for summer and winter seasons in the Rustfontein and Welbedacht dams. RRS represents Rustfontein Raw Summer, RRW represents Rustfontein Raw Winter, WRS represents Welbedacht Raw Summer, and WRW represents Welbedacht Raw Winter. The plot was generated using data generated from MetaPhlan (Supplementary Tables S6–S9).

Protocatechuate degradation (40%), anaerobic acetylene degradation (20%), dichloroethane degradation (11.4%), aerobic toluene degradation (2.7%), and the salicylate degradation super pathway (0.3%), among others, were detected in all four metagenomes (Figure 5). The latter three biodegradation pathways appeared to be more prevalent in winter.

### 3.5. The Presence of Genes Associated with the Emerging Contaminant Degradation

**Figure 6:**
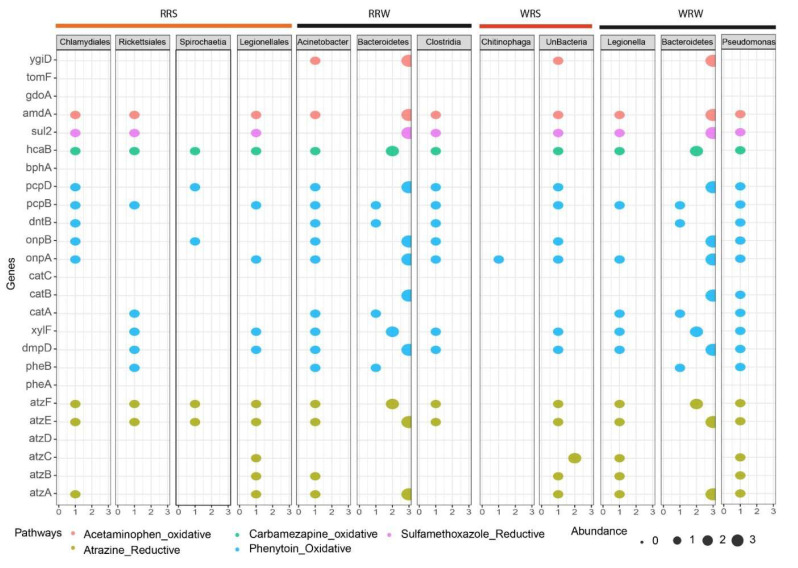
A bubble plot showing the presence of genes associated with emerging contaminant degradation and the bins identified in the Rustfontein and Welbedacht dams for the summer and winter seasons. The size range of the bubbles is 0–3, with 0 representing no abundance (not present) and 3 being the most abundant, generated using Supplementary Table S10. RRS represents Rustfontein Raw Summer, RRW represents Rustfontein Raw Winter, WRS represents Welbedacht Raw Summer, and WRW represents Welbedacht Raw Winter.

The quality of DNA extracted from these samples is crucial for subsequent analyses. Therefore, despite the observed seasonal differences in the relative abundance of genes and pathways, we must be cautious in interpreting these results, especially during the binning process, which is essential for assigning sequences to specific taxa. Consequently, no seasonal comparisons were conducted at the binning level; rather, the emphasis was on describing the potential genes identified in the metagenome-assembled genomes (MAGs) that are involved in degrading ECs. The MAGs (bacteria) assembled from the metagenomes of the wet season in the Rustfontein Dam were predominantly from the order Chlamydiales (95.92%) and Rickettsiales (95.24%), as well as the genus *Spirochaete* (80%) and *Legionellales* (81.49%). During the dry season, MAGs assembled from the metagenomes of the Rustfontein Dam belonged to the genus *Actinobacter* (79.29%), phylum Bacteroidetes (89.52%), and class Clostridia (96.55%). In the wet season at Welbedacht Dam, MAGs from the family Chitinophagaceae (76.67%) and cyanobacteria (85.74%) were assembled, while in the dry season, MAGs belonged to the genus *Legionella* (90.35%), phylum Bacteroidetes (81.88%), and genus *Pseudomonas* (90.2%) (Figure 6).

The MAGs isolated from the metagenomes of Rustfontein Dam demonstrated the potential to degrade atrazine (*atzA, atzB, atzC, atzD, and atzF*), phenytoin (*pheB, dmpD, xylF, catA, catC, onpA, onpB, dntB, pcpB, pcpD*), carbamazepine (*hcaB, bphA*), sulfamethoxazole (*sul2*), and acetaminophen (*amdA* and *ydiD*). Genes responsible for the degradation of all these ECs were also found in the MAGs from the metagenomes of Welbedacht Dam, except in Chitinophaga, which contained only one gene (*onpA*) associated with phenytoin degradation. The phylum Bacteroidetes appears to be well-equipped with genes involved in EC degradation.

## 4. Discussion

### 4.1. Physico-Chemical Characterization

Most of the physicochemical parameters analyzed in this study fell within established standard limits. For example, electrical conductivity values ranged between 17 and 42 mS/m across all seasons, which complies with the acceptable limits defined by SANS 241:2015, the South African Water Quality Guidelines for domestic use [43,44], and the World Health Organization (WHO) drinking water quality guidelines [45]. Dissolved oxygen (DO) concentrations varied from 4 to 12 mg/L; however, no formal standard currently exists for this parameter [45]. Dissolved organic carbon (DOC) concentrations, observed between 2 and 4 mg/L, also remained within the recommended SANS 241:2015 limits [43]. The pH values of the dams ranged within the recommended neutral range (≥5 to ≤9.7), consistent with SANS 214:2015 standards [43]. While no specific guidelines exist for oxidation-reduction potential (ORP/Eh), the concentrations of sulphate, nitrate, and ammonium all conformed to the standards set by SANS 214:2015, WHO, and South African Water Quality Guidelines [43,44,45].

Parameters such as temperature, pH, and oxygen are well-documented as influential factors affecting biodegradation rates by either limiting or promoting microbial activity [46]. Therefore, assessing these parameters was critical to determine whether metabolism or co-metabolism pathways could be enhanced within these aquatic systems. Temperature fluctuations, both high and low, have been shown to significantly affect microbial diversity, thereby impacting biodegradation efficiency [46,47]. Czajka and Londry (2006) reported that ammonia-oxidizing bacteria (AOB), such as *Nitrosomonas*, preferentially grow at lower temperatures, which promotes higher biodegradation rates [48]. This observation aligns with our data, where the abundance of co-metabolism-associated genes was higher in winter samples compared to summer.

pH also plays a vital role in microbial physiology, particularly influencing enzymatic activities critical for biodegradation [46]. Lower pH values have been linked to enhanced elimination of ECs, yet such acidic conditions can inhibit bacterial growth [46]. The near-neutral pH observed in our study likely favors bacterial proliferation, thereby facilitating biodegradation. DOC influences ECs transport by binding to these compounds, which affects their solubility, mobility, and ultimate biodegradation potential [49]. Oxidative conditions, as indicated by ORP and DO, stimulate monooxygenase enzymes known to enhance biodegradation [50]. Given these parameters and the relatively low concentrations of ECs compared to other substrates, it is plausible that co-metabolism represents the predominant metabolism pathway driving EC biodegradation in these dams [51,52].

### 4.2. Bacterial Diversity and Main Biogeochemical Pathways Associated with Co-Metabolism of ECs

Supporting this hypothesis, bacterial taxa belonging to the phyla Actinobacteria, Firmicutes, and Bacteroidetes, as well as genera such as *Pseudomonas* (Proteobacteria) and *Bacillus* (Firmicutes), were identified, which have been previously associated with co-metabolism degradation of persistent ECs, including polycyclic aromatic hydrocarbons and pesticides [53,54,55]. Notably, high removal efficiencies of pharmaceutical ECs have been linked to co-metabolism mediated by ammonia-oxidizing microorganisms utilizing ammonia monooxygenase (Amo) during nitrification, which acts as a precursor activating EC degradation [51]. In this study, the *amoABC* gene cluster, responsible for encoding Amo, was detected. Amo likely facilitates biodegradation through several biochemical reactions, including aliphatic and aromatic C-H hydroxylation, O-dealkylation, thioester oxidation, dehydrogenation, and nitration [51]. This enzyme has also been implicated in carbamazepine biodegradation via aromatic ring hydroxylation [51], a compound detected in the dam samples.

*Nitrosomonas*, *Nitrosospira*, and *Nitrospira* species were also identified, suggesting that indigenous bacteria capable of ammonia oxidation and co-metabolism are present. These bacteria are recognized for their roles in degrading ECs containing alkyl and benzene functional groups, especially in AOB-rich environments such as wastewater treatment plants [51,56].

Another co-metabolism enzyme of interest is methane monooxygenase (MMO). Methane-oxidizing bacteria (MOB), or methanotrophs, are capable of oxidizing a broad range of organic contaminants, including alkanes, alkenes, aromatic compounds, halogenated alkanes, and substituted aliphatics [57,58,59]. Methane-oxidizing bacteria express two forms of MMO: particulate membrane methane monooxygenase (pMMO) and soluble methane monooxygenase (sMMO) located in the cytoplasm [57]. Our samples contained the *mmoBCDXYZ* gene cluster encoding sMMO and *amoABC* encoding pMMO [60]. While most MOBs express pMMO, only a few express sMMO, which has broader substrate specificity and thus can co-metabolize a wider variety of ECs, enhancing bioremediation potential [57,59,61]. For instance, *Methylosinus trichosporium* has been reported to degrade up to 90% of sulfamethoxazole, likely via *pMMO*-mediated co-metabolism, though it exhibits lower degradation efficiency for atrazine and carbamazepine (~40%) [57]. Methylotrophs such as *Candidatus methylopumilus* and *Methylotenera*, members of the Methylophilaceae family frequently found in freshwater habitats, were also identified [62,63]. These bacteria have been linked to co-metabolism degradation of ECs like sulfamethoxazole and erythromycin, often through anaerobic methane oxidation coupled with nitrite reduction, further indicating their potential role in EC biodegradation [64].

Atrazine biodegradation may serve as an example of nitrogen cycling under co-metabolism conditions. Boopathy demonstrated that anaerobic biodegradation of atrazine occurs using nitrate and sulphate as electron acceptors, with removal efficiencies reaching up to 99% in the presence of molasses as a co-substrate [65]. Although ECs can be primary substrates in co-metabolism processes, their low environmental concentrations often necessitate the use of alternate carbon and nitrogen sources such as carbon dioxide and ammonia. The detection of sulphur-reducing activity in winter dam samples further supports the potential for co-metabolism processes in these systems. Gene abundance correlates with microbial diversity and physicochemical factors, including temperature. Previous studies have reported conflicting results regarding the impact of temperature on *amoA* gene abundance, a key gene in nitrification. Yang et al. observed higher *amoA* ratios in summer compared to winter [66], whereas Zheng et al. reported lower *amoA* abundance at higher temperatures [67], consistent with our Welbedacht samples. pH (~7.7) was neutral across samples and thus unlikely to negatively influence *amoA* abundance [68]. Similarly, low ammonium concentrations in our samples could explain the lower *amoA* gene abundance, contrasting with studies that link high ammonium to increased *amoA* [69]. Expression of methane monooxygenase genes is highly copper-dependent; gene expression of the sMMO enzyme occurs at low copper concentrations, while gene expression for pMMO enzyme predominates at higher copper levels [57,61]. Our copper levels (0.006–0.0014 mg/L) likely favored sMMO expression, reflected by the prominence of the *mmoBCDXYZ* cluster in dam samples. Optimal sMMO activity typically occurs at 20–25 °C and pH 6.0–7.0 [70,71,72], matching winter conditions in our study and indicating greater co-metabolism potential during colder months.

### 4.3. Presence of Xenobiotic Degradation Pathways and Genes Associated with Degradation in Relation to Seasonal Change

To validate the information obtained from the metagenome-assembled genomes (MAGs), we aimed to control the contamination and redundancy in the samples by selecting the best bins with a completeness greater than 75% and a contamination less than 5% to be used as the MAGs. All the bins selected to represent the MAGs had high completeness, ranging from 76 to 96 (Table S2), making our selection process efficient enough to select high-quality MAGs for further analysis. But the manual method can be improved by using tools such as dRep to dereplicate the genes and retain the best quality bin for analysis. Comparative tools such as FastANI and Pyani can also be utilized to compare bins to select high-quality ones to be used as MAGs.

Interestingly, the presence of xenobiotic biodegradative pathways suggests the potential for direct metabolism of ECs, which are classified as xenobiotics [73]. Protocatechuate degradation, associated with the breakdown of recalcitrant ECs like pesticides and polycyclic aromatic hydrocarbons by species such as *Pseudomonas*, was identified [74]. Other pathways detected include anaerobic acetylene degradation, aerobic toluene degradation, and salicylate degradation, each targeting specific EC compounds. The detection of genes linked to these pathways within metagenome-assembled genomes (MAGs) supports direct metabolism activity. Microbial exposure to organic contaminants often induces expression of specific degradative genes. For example, the *atzA*-*F* gene cluster, encoding enzymes for complete atrazine mineralization to carbon dioxide and ammonia, was detected, along with *Pseudomonas* and *Acinetobacter*, known atrazine degraders, in dam samples [75]. Genes related to acetaminophen, sulfamethoxazole, and phenytoin degradation were also present, even when the corresponding contaminants were not detected, suggesting potential for degradation if introduced.

Acetaminophen degradation proceeds via hydroquinone formation catalyzed by amidohydrolases encoded by *amdA* genes. Genera such as *Bacillus*, *Pseudomonas*, *Acinetobacter*, and *Staphylococcus* contribute to this pathway [76,77]. Gentisate 1,2-dioxygenase (*gdoA*) and enzymes like phenol 2-monooxygenase (*tomF*) and DOPA 4,5-dioxygenase (*ygiD*) mediate further aromatic ring cleavage and hydroxylation [78,79]. Carbamazepine biodegradation involves enzymes like biphenyl 2,3-diol dioxygenase (*bphA*) and dihydrodiol dehydrogenase (*hcaB/bphB*), facilitating aromatic ring cleavage and transformation [80,81,82]. Sulfamethoxazole metabolism involves species including *Acinetobacter* and *Pseudomonas*, with the *sul2* gene encoding dihydropteroate synthase being a key marker [83,84]. Phenytoin biodegradation has been associated with bacteria such as *Pseudomonas, Acinetobacter, Rhodococcus,* and *Sphingomonas* [85]. Phenytoin degradation pathways involve initial hydroxylation by phenol hydroxylase (*pheA*), followed by catechol ring cleavage via either ortho (*catA-C*) or meta (*pheB-D*) pathways, producing intermediates that feed into the TCA cycle [85,86]. Derivatives like 2-nitrophenol are degraded by enzymes 2-nitrophenol monooxygenase (*onpA*) and 1,2-Benzoquinone reductase (*onpB*), while pentachlorophenol hydroxylase (*pcpB*) and tetrachlorobenzoquinone reductase (*pcpC*) participate in aerobic degradation of related phenols [85,86]. Ultimately, these pathways channel EC breakdown products into central metabolism routes, enabling bacteria to utilize ECs as carbon sources.

### 4.4. Effect of Seasonal Change on the Degradation Efficiency and Ecological Implications of Finding Genes in Indigenous Bacteria

Environmental factors influenced by seasonal change can have a direct impact on metabolism degradation efficiency. For instance, higher temperatures have been linked to increased atrazine degradation [79,87], though our results showed greater gene abundance related to atrazine degradation during winter. The neutral pH (~7.5) and higher dissolved oxygen in winter may facilitate this process. Additionally, low ammonium and nitrate concentrations could relieve repression of atrazine degradation, which is often limited when preferred nitrogen sources are abundant [88,89]. Carbamazepine and phenytoin degradation genes were more abundant in summer at Rustfontein Dam, suggesting seasonal variability in degradation potential. Optimal sulfamethoxazole degradation occurs around 25 °C and pH 7 [90], consistent with higher gene detection in summer samples. Overall, the coexistence of microorganisms capable of both metabolism and co-metabolism degradation, the presence of xenobiotic degradation pathways, and genes linked to both processes suggest that these mechanisms may simultaneously contribute to EC biodegradation. This challenges the earlier hypothesis favouring exclusive co-metabolism. Supporting this, Rios-Miguel et al. reported that continuous exposure to acetaminophen selects for bacteria such as *Pseudomonas* and *Bacillus* with diverse metabolism capacities enabling both metabolism and co-metabolism degradation [79]. The detection of aerobic toluene degradation pathways and enzymes like toluene monooxygenase, implicated in both direct metabolism and co-metabolism of compounds like trichloroethylene (TCE), further corroborates this duality [91]. Enzymes encoded by genes found in MAGs, including oxygenases, are known to participate in aromatic compound catabolism and xenobiotic breakdown, reinforcing the potential for simultaneous metabolism and co-metabolism degradation of ECs. Catechol degradation pathways, central intermediates in aromatic compound metabolism, were also prominent, suggesting their importance in energy generation from EC biodegradation. By detecting the genes associated with metabolism and co-metabolism in the indigenous bacteria within the samples, it can be assumed that these bacteria are able to thrive in environments that are rich in ECs. Due to the low concentrations of ECs, the capacity to perform both metabolism and co-metabolism can ensure that the bacteria are able to efficiently use these ECs as energy sources without having ecotoxicological effects. This allows for the potential to select specific bacteria that are able to perform both metabolism and co-metabolism to increase the EC biodegradation efficiency.

## 5. Conclusions

Emerging contaminants pose a threat to the aquatic environment. To overcome the challenges faced, several studies have suggested the use of bacteria to effectively remove ECs from aquatic environments. This occurs through the processes of metabolism and co-metabolism, driven by specific enzymes encoded by EC-degrading genes. Herein, we successfully characterized two dams (seasonally) in the Free State, South Africa. The parameters outlined in the study were found to promote metabolism and co-metabolism during both seasons. The identification of bacteria such as *Pseudomonas*, *Acinetobacter*, and *Bacillus*, which are linked to metabolism and co-metabolism processes for the removal of emerging contaminants, supports the hypothesis that these processes can occur in both seasons. The study identified biogeochemical pathways that enabled the identification and quantification of gene clusters associated with ammonia monooxygenase and methane monooxygenase. Both these enzymes have been associated with the co-metabolism process of ECs and were present in both summer and winter, suggesting co-metabolism may occur during both seasons. The results were corroborated by the identification of ammonia-oxidizing bacteria such as *Nitrosmonas* and *Nitrosospira* and methylotrophs such as *Methylotenera* and *Candidatus* methylopumilus, which have been associated with EC biodegradation. The abundance of these genes was then evaluated in the MAGs, where some of the genes with direct metabolism were present. It can also be proposed that co-metabolism and metabolism may coexist in these aquatic systems, as evidenced by the presence of bacteria such as *Bacillus*, *Pseudomonas*, *Acinetobacter*, and *Staphylococcus*. Members of these bacterial genera encode enzymes like gentisate 1,2-dioxygenase, phenol hydroxylase (*pheA*), and amidohydrolase, as well as pathways involved in catechol and protocatechuate degradation, all of which have been linked to the breakdown of xenobiotics and aromatic emerging contaminants. Altogether, it can be concluded that the Indigenous microorganisms in the samples may have the potential to attenuate ECs in aquatic environments naturally through metabolism and co-metabolism regardless of the season.

## Figures and Tables

**Table 1:** Concentrations of emerging contaminants during summer and winter in the Modder River catchment used to represent the Rustfontein and Welbedacht dams.

Emerging Contaminant	Summer	Winter
Carbamazepine (ug/L)	0.21	0.19
Atrazine (mg/L)	0.04	0.02
Metolachlor (mg/L)	0.04	0.02
Terbuthylazine (mg/L)	0.08	0.03
17-alpha-ethinyl-estradiol (mg/L)	3.40	14.80

**Table 2:** Summary of physicochemical characteristics and elemental composition during summer and winter. The parameters are as follows: Eh: Oxidative Reductive Potential (mV), pH, EC: Electrical conductivity (mS/m), DO: Dissolved Oxygen (mg/L), DOC: Dissolved Organic Carbon (mg/L), SO_4_^2−^: Sulphate (mg/L), Nitrate: NO_3_^−^ (mg/L), Ammonium: NH_4_^+^ (mg/L), and Cu (mg/L).

Physicochemical Parameters									
	pH	Eh (mV)	EC (mS/m)	DO (mg/L)	DOC (mg/L)	SO_4_^2−^ (mg/L)	NO_3_^−^ (mg/L)	NH_4_^+^ (mg/L)	Cu (mg/L)
Summer									
Rustfontein Dam	7.83	202.50	17.81	8.00	2.71	8.99	0.44	0.18	0.07
Weldebacht Dam	7.58	176.30	42.61	4.00	3.62	19.93	0.29	0.20	0.006
Winter									
Rustfontein Dam	7.72	206.50	16.45	12.50	2.80	7.99	0.43	<0.10	0.053
Weldebacht Dam	7.58	160.50	40.61	5.00	4.20	20.13	0.30	<0.10	0.014

## Data Availability

The original contributions presented in this study are included in the article/Supplementary Materials. Further inquiries can be directed to the corresponding author.

## Supplementary Materials

The following supporting information can be downloaded at: https://www.mdpi.com/article/10.3390/microorganisms13102349/s1, Table S1. DNA, final library concentration and average library size. Figure S1. The boxplots showing the DNA concentrations quantified for three extraction methods: CTQ-CTAB, CTTBQ-CTAB-Tanbead method and TBQ-Tanbead method for the summer and winter samples in the Rustfontein and Welbedacht dams. Table S2. Relative abundance of Bacteria found in the Rustfontein Raw Summer samples generated from MetaPhlan used to create Figure 1. Table S3. Relative abundance of Bacteria found in the Rustfontein Raw Winter samples generated from MetaPhlan used to create Figure 1. Table S4. Relative abundance of Bacteria found in the Welbedacht Raw Summer samples generated from MetaPhlan used to create Figure 1. Table S5. Relative abundance of Bacteria found in the Welbedacht Raw Winter samples generated from MetaPhlan used to create Figure 1. Table S6. Presence of Xenobiotic pathways in the Rustfontein Raw Summer samples, used to generate Figure 5. Table S7. Presence of Xenobiotic Pathways in the Rustfontein Raw Winter samples, used to generate Figure 5. Table S8. Presence of Xenobiotic Pathways in the Welbedacht Raw Summer samples, used to generate Figure 5. Table S9. Presence of Xenobiotic Pathways in the Welbedacht Raw Winter samples used to generate Figure 5. Table S10. Bins with genes associated with emerging contaminants used to generate Figure 6 bubble plot.
