# Supplementary material for: First Insight into the Natural Attenuation of Emerging Contaminants Using a Metagenomics Approach from Drinking Water Sources in the Free State
Source: Microorganisms. 2025 Oct 14;13(10):2349. doi: 10.3390/microorganisms13102349 (PMC12565995; doi:10.3390/microorganisms13102349)
Supplement: Supplementary file 1 [file microorganisms-13-02349-s001.zip › microorganisms-3481843-supplementary.pdf]

microorganisms

MDPI

Supplementary materials

First Insight into the Natural Attenuation of Emerging Contaminants Using a Metagenomics Approach from Drinking Water Sources in the Free State

Avela Mqambalala [1], Maleke Maleke [2], Lore-Mari Deysel [3], Jorge R. Osman [4], Alba Gomez-Arias [5], Angel Valverde [6] and Julio Castillo Hernandez [1,7,*]

[1] Department of Microbiology and Biochemistry, University of the Free State, Bloemfontein 9300, South Africa; avelamqambalala@gmail.com
[2] Department of Life Sciences, Central University of Technology, Free State, Bloemfontein 9300, South Africa; mmaleke@cut.ac.za
[3] Institute of Groundwater Studies, University of the Free State, Bloemfontein 9300, South Africa; cruywagenlm@ufs.ac.za
[4] Instituto de Geología Económica Aplicada, Universidad de Concepción, Concepción 4070386, Chile; osman.jorge@gmail.com
[5] Instituto de Recursos Naturales y Agrobiología de Sevilla (IRNAS-CSIC), 41012 Sevilla, Spain; albita.anortita@gmail.com
[6] Instituto de Recursos Naturales y Agrobiologıa de Salamanca (IRNASA-CSIC), 37008 Salamanca, Spain; avalverdeportal@gmail.com
[7] Department of Integrated Science, University of Huelva, 21007 Huelva, Spain
* Correspondence: jccash007@gmail.com

Table S1. DNA, final library concentration and average library size.

Sample ID	DNA concentration (ng/µL)*	Final library DNA concentration (ng/µL)	Average Library size (bp)
**RRS**	119.0	31.4	1046
**RRW**	151.0	35.4	922
**WRS**	15.6	19.1	1075
**WRW**	135.0	19.5	926

*DNA concentration after whole genome amplification.

Optimization of DNA extraction

DNA was successfully extracted in all of the 12 samples for the Rustfontein and Welbedacht summer and winter samples (0.153–6.78ng/µL). Three methods were used for the DNA extraction. The CTAB method, TanBead method and CTAB–Tanbead method. Higher DNA concentrations were obtained after using the CTAB–Tanbead as compared to the CTAB and TanBead extraction methods. The CTAB–Tanbead method used is described as follows. Briefly, filter paper pieces were placed in 2 ml Eppendorf tubes. 500 µl TES Buffer (10 mM EDTA, 2% SDS, 100 mM Tris, pH 8) and 0.5 µl of glass beads were added. The tubes were then vortexed, incubated for 3 min at 100 °C, and on ice for 10 min. Thereafter, 1 µl of Proteinase K was added. The tubes were vortexed and incubated for 60 min at 60 °C. Then, 140 µL of NaCl (5 M) and 65 µl of 10% CTAB were added. The tubes were incubated at 60°C for 10 min. 700 µl was added and incubated on ice for 30 min. The tubes were centrifuged for 10 min at 12,000 rpm at 4 °C. The Assembled Auto Tubes were prepared by inserting them into the base of the Maelstrom 8 machine. The supernatant (lysate) from the CTAB

method was transferred to well #1 of the TAN Bead autotubes after removing the lysis buffer from the well. On the M8-H, the G1G-1 program was selected for the extraction.

There were no significant differences between the DNA concentrations obtained between the samples for both dams for all the methods used, as shown by the *p*-values obtained: CTAB method (0.562), CTAB–Tanbead method (0.181), and the TanBead method (0.757) respectively. As shown by the ANOVA test *p*-value (0.4841), there were also no significant differences in the DNA concentrations obtained between the methods used for the DNA extraction

.

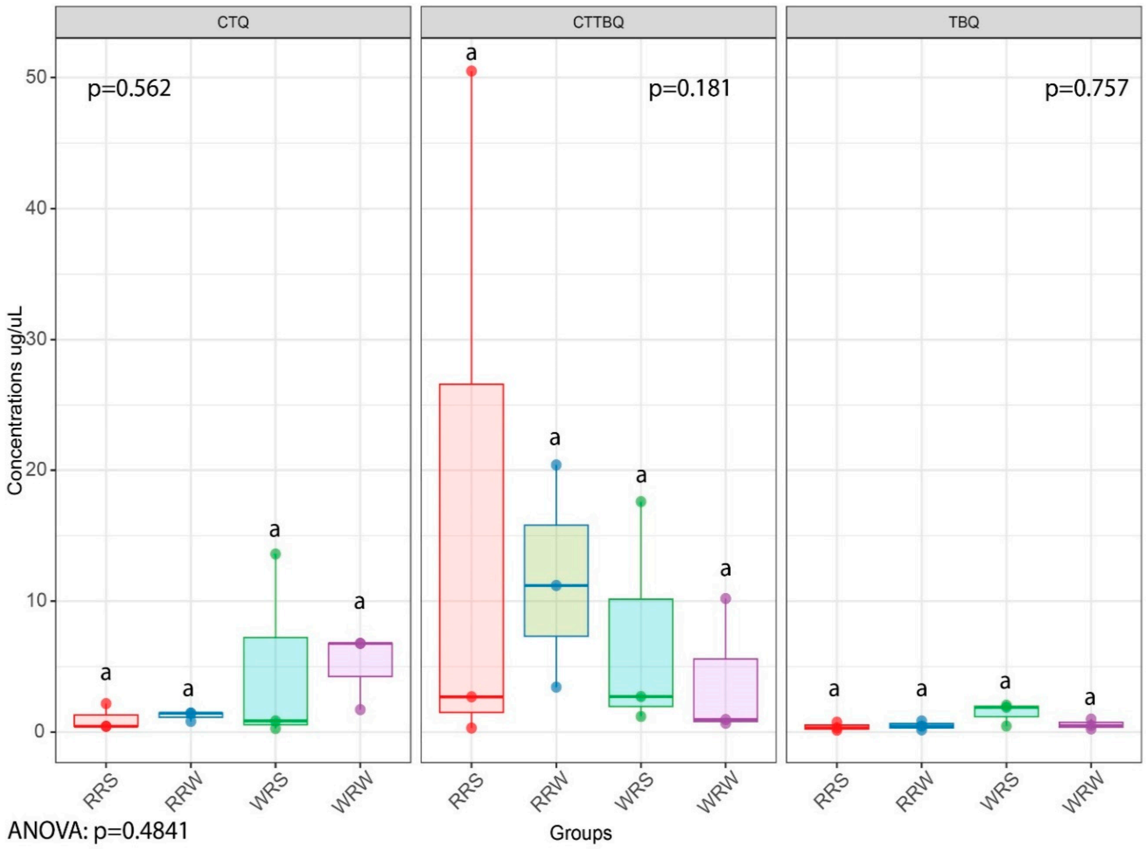

Figure S1. The boxplots showing the DNA concentrations quantified for three extraction methods: CTQ-CTAB, CTTBQ-CTAB-Tanbead method and TBQ-Tanbead method for the summer and winter samples in the Rustfontein and Welbedacht dams.

Site selection and Sampling

The raw surface water was collected from the large containers where the water is first pumped before being transferred into reservoirs. Then, the water is transferred to the influent tank for purification. Water samples for the summer season were collected on the 17th of March 2021 for Rustfontein Dam, and 21st of March 2021 for Welbedacht Dam. For the winter samples, the raw water was collected on the 26th and 27th of July 2021 for Rustfontein Dam and Welbedacht Dam, respectively.

Physiochemical analysis

The pH meter was calibrated at pH 1, 4, and 7 to ensure accurate measurements following the Standard Test Methods for the pH of water (ASTM D1293-12, Standard Test Method for pH of water).

Environmental genomic DNA Extraction

The G1G-1 program was as follows: The magnetic beads in well #3 were mixed at 3,000 rpm for 60 sec at 55 °C. The magnetic beads were then collected for 30 sec at 55 °C.

The washing buffer in well #2 was mixed at 3,000 rpm for 60 sec at 55 °C. The lysate in well #1 was mixed at 3,000 rpm for 1,200 sec at 55 °C. The washing buffer in well #2 was collected for 30 sec and mixed with the lysate in well #1 at 3,000 rpm for 600 sec at 55 °C. The lysate and washing buffer mix in well #1 was collected for 30 sec and mixed in well # 2 at 3,000 rpm for 120 sec at 45 °C. The lysate and washing buffer mix in well #2 was collected for 30 sec and mixed with the magnetic beads in well #3 at 3,000 rpm for 120 sec at 45 °C. The magnetic beads were collected for 30 sec and mixed with washing buffer 2 in well #4 at 3,000 rpm for 120 sec at 45 °C. The magnetic beads were collected for 30 sec and mixed in washing buffer in well #5 at 3,000 rpm for 120 sec 45 °C for a second wash. The magnetic beads were collected for 30 sec and vaporized for 600 sec and mixed in the elution buffer in well #6 at 3,000 rpm for 300 sec at 45 °C. The magnetic beads were collected for 30 sec and mixed with washing buffer 2 in well #5 at 3,000 rpm for 30 sec. The extraction was done in triplicates and the eluted DNA was pooled into 1.5 mL Eppendorf tubes. The pooled genomic DNA was dried and concentrated using the Speedy Vac for 30 min at 35 °C.

Table S2. Relative abundance of Bacteria found in the Rustfontein Raw Summer samples generated from MetaPhlan used to create Figure 1.

#SampleID	Metaphlan_Analysis	
#clade_name	NCBI_tax_id	relative_abundance
k__Bacteria	2	100.0
k__Bacteria|p__Proteobacteria	2|1224	38.03042
k__Bacteria|p__Acidobacteria	2|57723	34.52411
k__Bacteria|p__Actinobacteria	2|201174	18.03755
k__Bacteria|p__Bacteroidetes	2|976	9.38563
k__Bacteria|p__Fusobacteria	2|32066	0.02228
k__Bacteria|p__Acidobacteria|c__CFGB13989	2|57723|	34.52411
k__Bacteria|p__Proteobacteria|c__Betaproteobacteria	2|1224|28216	17.0721
k__Bacteria|p__Proteobacteria|c__Alphaproteobacteria	2|1224|28211	14.06314
k__Bacteria|p__Actinobacteria|c__CFGB34754	2|201174|	9.98741
k__Bacteria|p__Bacteroidetes|c__CFGB18754	2|976|	8.90589
k__Bacteria|p__Proteobacteria|c__Gammaproteobacteria	2|1224|1236	6.89519
k__Bacteria|p__Actinobacteria|c__Actinomycetia	2|201174|1760	6.07637
k__Bacteria|p__Actinobacteria|c__CFGB1083	2|201174|	1.92749
k__Bacteria|p__Bacteroidetes|c__CFGB76561	2|976|	0.45415
k__Bacteria|p__Actinobacteria|c__CFGB13659	2|201174|	0.04628
k__Bacteria|p__Fusobacteria|c__Fusobacteriia	2|32066|203490	0.02228
k__Bacteria|p__Bacteroidetes|c__CFGB35942	2|976|	0.02054
k__Bacteria|p__Bacteroidetes|c__CFGB41865	2|976|	0.00505
k__Bacteria|p__Acidobacteria|c__CFGB13989|o__OFGB13989	2|57723||	34.52411
k__Bacteria|p__Proteobacteria|c__Betaproteobacteria|o__Nitrosomonadales	2|1224|28216|32003	16.05897
k__Bacteria|p__Proteobacteria|c__Alphaproteobacteria|o__Pelagibacterales	2|1224|28211|54526	13.78276
k__Bacteria|p__Actinobacteria|c__CFGB34754|o__OFGB34754	2|201174||	9.98741
k__Bacteria|p__Bacteroidetes|c__CFGB18754|o__OFGB18754	2|976||	8.90589
k__Bacteria|p__Proteobacteria|c__Gammaproteobacteria|o__Pseudomonadales	2|1224|1236|72274	6.89519
k__Bacteria|p__Actinobacteria|c__Actinomycetia|o__Actinomycetia_unclassified	2|201174|1760|	5.18613
k__Bacteria|p__Actinobacteria|c__CFGB1083|o__OFGB1083	2|201174||	1.92749

**k__Bacteria|p__Proteobacteria|c__Betaproteobacteria|o__Burkholderiales**	2|1224|28216|80840	1.01313
**k__Bacteria|p__Actinobacteria|c__Actinomycetia|o__Candidatus_Nanopelagicales**	2|201174|1760|2039638	0.79759
**k__Bacteria|p__Bacteroidetes|c__CFGB76561|o__OFGB76561**	2|976||	0.45415
**k__Bacteria|p__Proteobacteria|c__Alphaproteobacteria|o__Alphaproteobacteria_unclassified**	2|1224|28211|	0.28038
**k__Bacteria|p__Actinobacteria|c__Actinomycetia|o__Bifidobacteriales**	2|201174|1760|85004	0.09265
**k__Bacteria|p__Actinobacteria|c__CFGB13659|o__OFGB13659**	2|201174||	0.04628
**k__Bacteria|p__Fusobacteria|c__Fusobacteriia|o__Fusobacteriales**	2|32066|203490|203491	0.02228
**k__Bacteria|p__Bacteroidetes|c__CFGB35942|o__OFGB35942**	2|976||	0.02054
**k__Bacteria|p__Bacteroidetes|c__CFGB41865|o__OFGB41865**	2|976||	0.00505
**k__Bacteria|p__Acidobacteria|c__CFGB13989|o__OFGB13989|f__FGB13989**	2|57723|||	34.52411
**k__Bacteria|p__Proteobacteria|c__Betaproteobacteria|o__Nitrosomonadales|f__Methylophilaceae**	2|1224|28216|32003|32011	15.98456
**k__Bacteria|p__Proteobacteria|c__Alphaproteobacteria|o__Pelagibacterales|f__Pelagibacterales_unclassified**	2|1224|28211|54526|	13.78276
**k__Bacteria|p__Actinobacteria|c__CFGB34754|o__OFGB34754|f__FGB34754**	2|201174|||	9.98741
**k__Bacteria|p__Bacteroidetes|c__CFGB18754|o__OFGB18754|f__FGB18754**	2|976|||	8.90589
**k__Bacteria|p__Actinobacteria|c__Actinomycetia|o__Actinomycetia_unclassified|f__Actinomycetia_unclassified**	2|201174|1760||	5.18613
**k__Bacteria|p__Proteobacteria|c__Gammaproteobacteria|o__Pseudomonadales|f__Pseudomonadaceae**	2|1224|1236|72274|135621	5.00624
**k__Bacteria|p__Actinobacteria|c__CFGB1083|o__OFGB1083|f__FGB1083**	2|201174|||	1.92749
**k__Bacteria|p__Proteobacteria|c__Gammaproteobacteria|o__Pseudomonadales|f__Perlucidibacaceae**	2|1224|1236|72274|2887331	1.88895
**k__Bacteria|p__Proteobacteria|c__Betaproteobacteria|o__Burkholderiales|f__Burkholderiaceae**	2|1224|28216|80840|119060	1.01313
**k__Bacteria|p__Actinobacteria|c__Actinomycetia|o__Candidatus_Nanopelagicales|f__Candidatus_Nanopelagicaceae**	2|201174|1760|2039638|2162846	0.79759
**k__Bacteria|p__Bacteroidetes|c__CFGB76561|o__OFGB76561|f__FGB76561**	2|976|||	0.45415
**k__Bacteria|p__Proteobacteria|c__Alphaproteobacteria|o__Alphaproteobacteria_unclassified|f__Alphaproteobacteria_unclassified**	2|1224|28211||	0.28038
**k__Bacteria|p__Actinobacteria|c__Actinomycetia|o__Bifidobacteriales|f__Bifidobacteriaceae**	2|201174|1760|85004|31953	0.09265
**k__Bacteria|p__Proteobacteria|c__Betaproteobacteria|o__Nitrosomonadales|f__Nitrosomonadaceae**	2|1224|28216|32003|206379	0.07441
**k__Bacteria|p__Actinobacteria|c__CFGB13659|o__OFGB13659|f__FGB13659**	2|201174|||	0.04628
**k__Bacteria|p__Fusobacteria|c__Fusobacteriia|o__Fusobacteriales|f__Leptotrichiaceae**	2|32066|203490|203491|1129771	0.02228
**k__Bacteria|p__Bacteroidetes|c__CFGB35942|o__OFGB35942|f__FGB35942**	2|976|||	0.02054

**k__Bacteria|p__Bacteroidetes|c__CFGB41865|o__OFGB41865|f__FGB41865**	2|976|||	0.00505
**k__Bacteria|p__Acidobacteria|c__CFGB13989|o__OFGB13989|f__FGB13989|g__GGB74065**	2|57723||||	34.52411
**k__Bacteria|p__Proteobacteria|c__Betaproteobacteria|o__Nitrosomonadales|f__Methylophilaceae|g__Candidatus_Methylopumilus**	2|1224|28216|32003|32011|1679002	15.98456
**k__Bacteria|p__Proteobacteria|c__Alphaproteobacteria|o__Pelagibacterales|f__Pelagibacterales_unclassified|g__Candidatus_Fonsibacter**	2|1224|28211|54526||2045213	13.78276
**k__Bacteria|p__Actinobacteria|c__CFGB34754|o__OFGB34754|f__FGB34754|g__GGB34754**	2|201174||||	9.98741
**k__Bacteria|p__Bacteroidetes|c__CFGB18754|o__OFGB18754|f__FGB18754|g__GGB46527**	2|976||||	8.90589
**k__Bacteria|p__Actinobacteria|c__Actinomycetia|o__Actinomycetia_unclassified|f__Actinomycetia_unclassified|g__Actinomycetia_unclassified**	2|201174|1760|||	5.18613
**k__Bacteria|p__Proteobacteria|c__Gammaproteobacteria|o__Pseudomonadales|f__Pseudomonadaceae|g__Pseudomonas**	2|1224|1236|72274|135621|286	5.00624
**k__Bacteria|p__Actinobacteria|c__CFGB1083|o__OFGB1083|f__FGB1083|g__GGB24856**	2|201174||||	1.92749
**k__Bacteria|p__Proteobacteria|c__Gammaproteobacteria|o__Pseudomonadales|f__Perlucidibacaceae|g__Perlucidibaca**	2|1224|1236|72274|2887331|661182	1.88895
**k__Bacteria|p__Proteobacteria|c__Betaproteobacteria|o__Burkholderiales|f__Burkholderiaceae|g__GGB26028**	2|1224|28216|80840|119060|	1.01313
**k__Bacteria|p__Actinobacteria|c__Actinomycetia|o__Candidatus_Nanopelagicales|f__Candidatus_Nanopelagicaceae|g__Candidatus_Planktophila**	2|201174|1760|2039638|2162846|622681	0.51646
**k__Bacteria|p__Bacteroidetes|c__CFGB76561|o__OFGB76561|f__FGB76561|g__GGB46503**	2|976||||	0.45415
**k__Bacteria|p__Actinobacteria|c__Actinomycetia|o__Candidatus_Nanopelagicales|f__Candidatus_Nanopelagicaceae|g__Candidatus_Nanopelagicus**	2|201174|1760|2039638|2162846|2039639	0.28113
**k__Bacteria|p__Proteobacteria|c__Alphaproteobacteria|o__Alphaproteobacteria_unclassified|f__Alphaproteobacteria_unclassified|g__Alphaproteobacteria_unclassified**	2|1224|28211|||	0.28038
**k__Bacteria|p__Actinobacteria|c__Actinomycetia|o__Bifidobacteriales|f__Bifidobacteriaceae|g__Gardnerella**	2|201174|1760|85004|31953|2701	0.09265
**k__Bacteria|p__Proteobacteria|c__Betaproteobacteria|o__Nitrosomonadales|f__Nitrosomonadaceae|g__Nitrosospira**	2|1224|28216|32003|206379|35798	0.07441
**k__Bacteria|p__Actinobacteria|c__CFGB13659|o__OFGB13659|f__FGB13659|g__GGB23686**	2|201174||||	0.04628
**k__Bacteria|p__Fusobacteria|c__Fusobacteriia|o__Fusobacteriales|f__Leptotrichiaceae|g__Sneathia**	2|32066|203490|203491|1129771|168808	0.02228
**k__Bacteria|p__Bacteroidetes|c__CFGB35942|o__OFGB35942|f__FGB35942|g__GGB56722**	2|976||||	0.02054
**k__Bacteria|p__Bacteroidetes|c__CFGB41865|o__OFGB41865|f__FGB41865|g__GGB75267**	2|976||||	0.00505
**k__Bacteria|p__Acidobacteria|c__CFGB13989|o__OFGB13989|f__FGB13989|g__GGB74065|s__GGB74065_SGB52643**	2|57723|||||	34.52411
**k__Bacteria|p__Proteobacteria|c__Alphaproteobacteria|o__Pelagibacterales|f__Pelagibacterales_unclassified|g__Candidatus_Fonsibacter|s__Candidatus_Fonsibacter_ubiquis**	2|1224|28211|54526||2045213|1925548	13.78276

**k__Bacteria|p__Proteobacteria|c__Betaproteobacteria|o__Nitrosomonadales|f__Methylophilaceae|g__Candidatus_Methylopumilus|s__Candidatus_Methylopumilus_rimovensis**	2|1224|28216|32003|32011|1679002|2588535	10.61354
**k__Bacteria|p__Actinobacteria|c__CFGB34754|o__OFGB34754|f__FGB34754|g__GGB34754|s__GGB34754_SGB82226**	2|201174|||||	9.98741
**k__Bacteria|p__Bacteroidetes|c__CFGB18754|o__OFGB18754|f__FGB18754|g__GGB46527|s__GGB46527_SGB64388**	2|976|||||	8.90589
**k__Bacteria|p__Proteobacteria|c__Betaproteobacteria|o__Nitrosomonadales|f__Methylophilaceae|g__Candidatus_Methylopumilus|s__Candidatus_Methylopumilus_universalis**	2|1224|28216|32003|32011|1679002|2588536	5.37102
**k__Bacteria|p__Actinobacteria|c__Actinomycetia|o__Actinomycetia_unclassified|f__Actinomycetia_unclassified|g__Actinomycetia_unclassified|s__Actinomycetia_unclassified_SGB64102**	2|201174|1760||||	4.61117
**k__Bacteria|p__Proteobacteria|c__Gammaproteobacteria|o__Pseudomonadales|f__Pseudomonadaceae|g__Pseudomonas|s__Pseudomonas_sp_PDM05**	2|1224|1236|72274|135621|286|2769301	3.0833
**k__Bacteria|p__Actinobacteria|c__CFGB1083|o__OFGB1083|f__FGB1083|g__GGB24856|s__GGB24856_SGB81948**	2|201174|||||	1.92749
**k__Bacteria|p__Proteobacteria|c__Gammaproteobacteria|o__Pseudomonadales|f__Perlucidibacaceae|g__Perlucidibaca|s__Perlucidibaca_aquatica**	2|1224|1236|72274|2887331|661182|1852776	1.88895
**k__Bacteria|p__Proteobacteria|c__Betaproteobacteria|o__Burkholderiales|f__Burkholderiaceae|g__GGB26028|s__GGB26028_SGB38031**	2|1224|28216|80840|119060||	0.79151
**k__Bacteria|p__Proteobacteria|c__Gammaproteobacteria|o__Pseudomonadales|f__Pseudomonadaceae|g__Pseudomonas|s__Pseudomonas_viridiflava**	2|1224|1236|72274|135621|286|33069	0.72737
**k__Bacteria|p__Proteobacteria|c__Gammaproteobacteria|o__Pseudomonadales|f__Pseudomonadaceae|g__Pseudomonas|s__Pseudomonas_simiae**	2|1224|1236|72274|135621|286|321846	0.66698
**k__Bacteria|p__Actinobacteria|c__Actinomycetia|o__Actinomycetia_unclassified|f__Actinomycetia_unclassified|g__Actinomycetia_unclassified|s__actinobacterium_SCGC_AAA028_A23**	2|201174|1760||||932036	0.57496
**k__Bacteria|p__Bacteroidetes|c__CFGB76561|o__OFGB76561|f__FGB76561|g__GGB46503|s__GGB46503_SGB91068**	2|976|||||	0.45415
**k__Bacteria|p__Proteobacteria|c__Gammaproteobacteria|o__Pseudomonadales|f__Pseudomonadaceae|g__Pseudomonas|s__Pseudomonas_sp_CBZ_4**	2|1224|1236|72274|135621|286|1163065	0.43821
**k__Bacteria|p__Actinobacteria|c__Actinomycetia|o__Candidatus_Nanopelagicales|f__Candidatus_Nanopelagicaceae|g__Candidatus_Planktophila|s__Candidatus_Planktophila_vernalis**	2|201174|1760|2039638|2162846|622681|1884907	0.35171
**k__Bacteria|p__Proteobacteria|c__Alphaproteobacteria|o__Alphaproteobacteria_unclassified|f__Alphaproteobacteria_unclassified|g__Alphaproteobacteria_unclassified|s__alpha_proteobacterium_SCGC_AAA028_D10**	2|1224|28211||||938641	0.28038
**k__Bacteria|p__Actinobacteria|c__Actinomycetia|o__Candidatus_Nanopelagicales|f__Candidatus_Nanopelagicaceae|g__Candidatus_Nanopelagicus|s__Candidatus_Nanopelagicus_limnes**	2|201174|1760|2039638|2162846|2039639|1884634	0.22317
**k__Bacteria|p__Proteobacteria|c__Betaproteobacteria|o__Burkholderiales|f__Burkholderiaceae|g__GGB26028|s__GGB26028_SGB38032**	2|1224|28216|80840|119060||	0.22162
**k__Bacteria|p__Actinobacteria|c__Actinomycetia|o__Candidatus_Nanopelagicales|f__Candidatus_Nanopelagicaceae|g__Candidatus_Planktophila|s__Candidatus_Planktophila_lacus**	2|201174|1760|2039638|2162846|622681|1884913	0.16476
**k__Bacteria|p__Actinobacteria|c__Actinomycetia|o__Bifidobacteriales|f__Bifidobacteriaceae|g__Gardnerella|s__Gardnerella_vaginalis**	2|201174|1760|85004|31953|2701|2702	0.09265

**k__Bacteria|p__Proteobacteria|c__Gammaproteobacteria|o__Pseudomonadales|f__Pseudomonadaceae|g__Pseudomonas|s__Pseudomonas_fluorescens**	2|1224|1236|72274|135621|286|294	0.09037
**k__Bacteria|p__Proteobacteria|c__Betaproteobacteria|o__Nitrosomonadales|f__Nitrosomonadaceae|g__Nitrosospira|s__Nitrosospira_sp_Nsp13**	2|1224|28216|32003|206379|35798|1855332	0.07441
**k__Bacteria|p__Actinobacteria|c__Actinomycetia|o__Candidatus_Nanopelagicales|f__Candidatus_Nanopelagicaceae|g__Candidatus_Nanopelagicus|s__Candidatus_Nanopelagicus_abundans**	2|201174|1760|2039638|2162846|2039639|1884916	0.05796
**k__Bacteria|p__Actinobacteria|c__CFGB13659|o__OFGB13659|f__FGB13659|g__GGB23686|s__GGB23686_SGB82209**	2|201174|||||	0.04628
**k__Bacteria|p__Fusobacteria|c__Fusobacteriia|o__Fusobacteriales|f__Leptotrichiaceae|g__Sneathia|s__Sneathia_vaginalis**	2|32066|203490|203491|1129771|168808|187101	0.02228
**k__Bacteria|p__Bacteroidetes|c__CFGB35942|o__OFGB35942|f__FGB35942|g__GGB56722|s__GGB56722_SGB78156**	2|976|||||	0.02054
**k__Bacteria|p__Bacteroidetes|c__CFGB41865|o__OFGB41865|f__FGB41865|g__GGB75267|s__GGB75267_SGB103593**	2|976|||||	0.00505
**k__Bacteria|p__Acidobacteria|c__CFGB13989|o__OFGB13989|f__FGB13989|g__GGB74065|s__GGB74065_SGB52643|t__SGB52643**	2|57723||||||	34.52411
**k__Bacteria|p__Proteobacteria|c__Alphaproteobacteria|o__Pelagibacterales|f__Pelagibacterales_unclassified|g__Candidatus_Fonsibacter|s__Candidatus_Fonsibacter_ubiquis|t__SGB28829**	2|1224|28211|54526||2045213|1925548|	13.78276
**k__Bacteria|p__Proteobacteria|c__Betaproteobacteria|o__Nitrosomonadales|f__Methylophilaceae|g__Candidatus_Methylopumilus|s__Candidatus_Methylopumilus_rimovensis|t__SGB80878**	2|1224|28216|32003|32011|1679002|2588535|	10.61354
**k__Bacteria|p__Actinobacteria|c__CFGB34754|o__OFGB34754|f__FGB34754|g__GGB34754|s__GGB34754_SGB82226|t__SGB82226**	2|201174||||||	9.98741
**k__Bacteria|p__Bacteroidetes|c__CFGB18754|o__OFGB18754|f__FGB18754|g__GGB46527|s__GGB46527_SGB64388|t__SGB64388**	2|976||||||	8.90589
**k__Bacteria|p__Proteobacteria|c__Betaproteobacteria|o__Nitrosomonadales|f__Methylophilaceae|g__Candidatus_Methylopumilus|s__Candidatus_Methylopumilus_universalis|t__SGB38034**	2|1224|28216|32003|32011|1679002|2588536|	5.37102
**k__Bacteria|p__Actinobacteria|c__Actinomycetia|o__Actinomycetia_unclassified|f__Actinomycetia_unclassified|g__Actinomycetia_unclassified|s__Actinomycetia_unclassified_SGB64102|t__SGB64102**	2|201174|1760|||||	4.61117
**k__Bacteria|p__Proteobacteria|c__Gammaproteobacteria|o__Pseudomonadales|f__Pseudomonadaceae|g__Pseudomonas|s__Pseudomonas_sp_PDM05|t__SGB89067**	2|1224|1236|72274|135621|286|2769301|	3.0833
**k__Bacteria|p__Actinobacteria|c__CFGB1083|o__OFGB1083|f__FGB1083|g__GGB24856|s__GGB24856_SGB81948|t__SGB81948**	2|201174||||||	1.92749
**k__Bacteria|p__Proteobacteria|c__Gammaproteobacteria|o__Pseudomonadales|f__Perlucidibacaceae|g__Perlucidibaca|s__Perlucidibaca_aquatica|t__SGB33026**	2|1224|1236|72274|2887331|661182|1852776|	1.88895
**k__Bacteria|p__Proteobacteria|c__Betaproteobacteria|o__Burkholderiales|f__Burkholderiaceae|g__GGB26028|s__GGB26028_SGB38031|t__SGB38031**	2|1224|28216|80840|119060|||	0.79151
**k__Bacteria|p__Proteobacteria|c__Gammaproteobacteria|o__Pseudomonadales|f__Pseudomonadaceae|g__Pseudomonas|s__Pseudomonas_viridiflava|t__SGB12132**	2|1224|1236|72274|135621|286|33069|	0.72737
**k__Bacteria|p__Proteobacteria|c__Gammaproteobacteria|o__Pseudomonadales|f__Pseudomonadaceae|g__Pseudomonas|s__Pseudomonas_simiae|t__SGB12145**	2|1224|1236|72274|135621|286|321846|	0.66698

<table>
<tr><td>k__Bacteria|p__Actinobacteria|c__Actinomycetia|o__Actinomycetia_unclassified|f__Actinomycetia_unclassified|g__Actinomycetia_unclassified|s__actinobacterium_SCGC_AAA028_A23|t__SGB5713</td><td>2|201174|1760||||932036|</td><td>0.57496</td></tr>
<tr><td>k__Bacteria|p__Bacteroidetes|c__CFGB76561|o__OFGB76561|f__FGB76561|g__GGB46503|s__GGB46503_SGB91068|t__SGB91068</td><td>2|976||||||</td><td>0.45415</td></tr>
<tr><td>k__Bacteria|p__Proteobacteria|c__Gammaproteobacteria|o__Pseudomonadales|f__Pseudomonadaceae|g__Pseudomonas|s__Pseudomonas_sp_CBZ_4|t__SGB33231</td><td>2|1224|1236|72274|135621|286|1163065|</td><td>0.43821</td></tr>
<tr><td>k__Bacteria|p__Actinobacteria|c__Actinomycetia|o__Candidatus_Nanopelagicales|f__Candidatus_Nanopelagicaceae|g__Candidatus_Planktophila|s__Candidatus_Planktophila_vernalis|t__SGB5705</td><td>2|201174|1760|2039638|2162846|622681|1884907|</td><td>0.35171</td></tr>
<tr><td>k__Bacteria|p__Proteobacteria|c__Alphaproteobacteria|o__Alphaproteobacteria_unclassified|f__Alphaproteobacteria_unclassified|g__Alphaproteobacteria_unclassified|s__alpha_proteobacterium_SCGC_AAA028_D10|t__SGB50895_group</td><td>2|1224|28211||||938641|</td><td>0.28038</td></tr>
<tr><td>k__Bacteria|p__Actinobacteria|c__Actinomycetia|o__Candidatus_Nanopelagicales|f__Candidatus_Nanopelagicaceae|g__Candidatus_Nanopelagicus|s__Candidatus_Nanopelagicus_limnes|t__SGB5711</td><td>2|201174|1760|2039638|2162846|2039639|1884634|</td><td>0.22317</td></tr>
<tr><td>k__Bacteria|p__Proteobacteria|c__Betaproteobacteria|o__Burkholderiales|f__Burkholderiaceae|g__GGB26028|s__GGB26028_SGB38032|t__SGB38032</td><td>2|1224|28216|80840|119060|||</td><td>0.22162</td></tr>
<tr><td>k__Bacteria|p__Actinobacteria|c__Actinomycetia|o__Candidatus_Nanopelagicales|f__Candidatus_Nanopelagicaceae|g__Candidatus_Planktophila|s__Candidatus_Planktophila_lacus|t__SGB5706</td><td>2|201174|1760|2039638|2162846|622681|1884913|</td><td>0.16476</td></tr>
<tr><td>k__Bacteria|p__Actinobacteria|c__Actinomycetia|o__Bifidobacteriales|f__Bifidobacteriaceae|g__Gardnerella|s__Gardnerella_vaginalis|t__SGB17307</td><td>2|201174|1760|85004|31953|2701|2702|</td><td>0.09265</td></tr>
<tr><td>k__Bacteria|p__Proteobacteria|c__Gammaproteobacteria|o__Pseudomonadales|f__Pseudomonadaceae|g__Pseudomonas|s__Pseudomonas_fluorescens|t__SGB12163</td><td>2|1224|1236|72274|135621|286|294|</td><td>0.09037</td></tr>
<tr><td>k__Bacteria|p__Proteobacteria|c__Betaproteobacteria|o__Nitrosomonadales|f__Nitrosomonadaceae|g__Nitrosospira|s__Nitrosospira_sp_Nsp13|t__SGB13341</td><td>2|1224|28216|32003|206379|35798|1855332|</td><td>0.07441</td></tr>
<tr><td>k__Bacteria|p__Actinobacteria|c__Actinomycetia|o__Candidatus_Nanopelagicales|f__Candidatus_Nanopelagicaceae|g__Candidatus_Nanopelagicus|s__Candidatus_Nanopelagicus_abundans|t__SGB5714</td><td>2|201174|1760|2039638|2162846|2039639|1884916|</td><td>0.05796</td></tr>
<tr><td>k__Bacteria|p__Actinobacteria|c__CFGB13659|o__OFGB13659|f__FGB13659|g__GGB23686|s__GGB23686_SGB82209|t__SGB82209</td><td>2|201174||||||</td><td>0.04628</td></tr>
<tr><td>k__Bacteria|p__Fusobacteria|c__Fusobacteriia|o__Fusobacteriales|f__Leptotrichiaceae|g__Sneathia|s__Sneathia_vaginalis|t__SGB6044</td><td>2|32066|203490|203491|1129771|168808|187101|</td><td>0.02228</td></tr>
<tr><td>k__Bacteria|p__Bacteroidetes|c__CFGB35942|o__OFGB35942|f__FGB35942|g__GGB56722|s__GGB56722_SGB78156|t__SGB78156</td><td>2|976||||||</td><td>0.02054</td></tr>
<tr><td>k__Bacteria|p__Bacteroidetes|c__CFGB41865|o__OFGB41865|f__FGB41865|g__GGB75267|s__GGB75267_SGB103593|t__SGB103593</td><td>2|976||||||</td><td>0.00505</td></tr>
</table>

Table S3. Relative abundance of Bacteria found in the Rustfontein Raw Winter samples generated from MetaPhlan used to create Figure 1.

#SampleID	Metaphlan_Analysis

#clade_name	NCBI_tax_id	relative_abundance
**k__Bacteria**	2	100.0
**k__Bacteria|p__Proteobacteria**	2|1224	54.86397
**k__Bacteria|p__Acidobacteria**	2|57723	23.00169
**k__Bacteria|p__Actinobacteria**	2|201174	13.95404
**k__Bacteria|p__Bacteroidetes**	2|976	6.62349
**k__Bacteria|p__Firmicutes**	2|1239	1.55681
**k__Bacteria|p__Proteobacteria|c__Gammaproteobacteria**	2|1224|1236	25.59295
**k__Bacteria|p__Acidobacteria|c__CFGB13989**	2|57723|	23.00169
**k__Bacteria|p__Proteobacteria|c__Alphaproteobacteria**	2|1224|28211	18.93325
**k__Bacteria|p__Proteobacteria|c__Betaproteobacteria**	2|1224|28216	10.28914
**k__Bacteria|p__Actinobacteria|c__CFGB34754**	2|201174|	7.09333
**k__Bacteria|p__Bacteroidetes|c__CFGB18754**	2|976|	6.58054
**k__Bacteria|p__Actinobacteria|c__Actinomycetia**	2|201174|1760	4.14037
**k__Bacteria|p__Actinobacteria|c__CFGB1083**	2|201174|	2.5097
**k__Bacteria|p__Firmicutes|c__Bacilli**	2|1239|91061	1.55681
**k__Bacteria|p__Actinobacteria|c__CFGB13153**	2|201174|	0.13493
**k__Bacteria|p__Actinobacteria|c__CFGB13659**	2|201174|	0.07571
**k__Bacteria|p__Proteobacteria|c__CFGB76227**	2|1224|	0.04863
**k__Bacteria|p__Bacteroidetes|c__CFGB14294**	2|976|	0.04295
**k__Bacteria|p__Acidobacteria|c__CFGB13989|o__OFGB13989**	2|57723||	23.00169
**k__Bacteria|p__Proteobacteria|c__Gammaproteobacteria|o__Moraxellales**	2|1224|1236|2887326	19.55144
**k__Bacteria|p__Proteobacteria|c__Alphaproteobacteria|o__Pelagibacterales**	2|1224|28211|54526	17.07819
**k__Bacteria|p__Proteobacteria|c__Betaproteobacteria|o__Nitrosomonadales**	2|1224|28216|32003	9.49904
**k__Bacteria|p__Actinobacteria|c__CFGB34754|o__OFGB34754**	2|201174||	7.09333
**k__Bacteria|p__Bacteroidetes|c__CFGB18754|o__OFGB18754**	2|976||	6.58054
**k__Bacteria|p__Proteobacteria|c__Gammaproteobacteria|o__Pseudomonadales**	2|1224|1236|72274	6.0415
**k__Bacteria|p__Actinobacteria|c__Actinomycetia|o__Actinomycetia_unclassified**	2|201174|1760|	3.65623
**k__Bacteria|p__Actinobacteria|c__CFGB1083|o__OFGB1083**	2|201174||	2.5097
**k__Bacteria|p__Proteobacteria|c__Alphaproteobacteria|o__Alphaproteobacteria_unclassified**	2|1224|28211|	1.85506

**k__Bacteria|p__Firmicutes|c__Bacilli|o__Bacillales**	2|1239|91061|1385	1.55681
**k__Bacteria|p__Proteobacteria|c__Betaproteobacteria|o__Burkholderiales**	2|1224|28216|80840	0.73303
**k__Bacteria|p__Actinobacteria|c__Actinomycetia|o__Candidatus_Nanopelagicales**	2|201174|1760|2039638	0.48415
**k__Bacteria|p__Actinobacteria|c__CFGB13153|o__OFGB13153**	2|201174||	0.13493
**k__Bacteria|p__Actinobacteria|c__CFGB13659|o__OFGB13659**	2|201174||	0.07571
**k__Bacteria|p__Proteobacteria|c__Betaproteobacteria|o__Rhodocyclales**	2|1224|28216|206389	0.05707
**k__Bacteria|p__Proteobacteria|c__CFGB76227|o__OFGB76227**	2|1224||	0.04863
**k__Bacteria|p__Bacteroidetes|c__CFGB14294|o__OFGB14294**	2|976||	0.04295
**k__Bacteria|p__Acidobacteria|c__CFGB13989|o__OFGB13989|f__FGB13989**	2|57723|||	23.00169
**k__Bacteria|p__Proteobacteria|c__Gammaproteobacteria|o__Moraxellales|f__Moraxellaceae**	2|1224|1236|2887326|468	19.55144
**k__Bacteria|p__Proteobacteria|c__Alphaproteobacteria|o__Pelagibacterales|f__Pelagibacterales_unclassified**	2|1224|28211|54526|	17.07819
**k__Bacteria|p__Proteobacteria|c__Betaproteobacteria|o__Nitrosomonadales|f__Methylophilaceae**	2|1224|28216|32003|32011	9.49904
**k__Bacteria|p__Actinobacteria|c__CFGB34754|o__OFGB34754|f__FGB34754**	2|201174|||	7.09333
**k__Bacteria|p__Bacteroidetes|c__CFGB18754|o__OFGB18754|f__FGB18754**	2|976|||	6.58054
**k__Bacteria|p__Proteobacteria|c__Gammaproteobacteria|o__Pseudomonadales|f__Pseudomonadaceae**	2|1224|1236|72274|135621	5.34458
**k__Bacteria|p__Actinobacteria|c__Actinomycetia|o__Actinomycetia_unclassified|f__Actinomycetia_unclassified**	2|201174|1760||	3.65623
**k__Bacteria|p__Actinobacteria|c__CFGB1083|o__OFGB1083|f__FGB1083**	2|201174|||	2.5097
**k__Bacteria|p__Proteobacteria|c__Alphaproteobacteria|o__Alphaproteobacteria_unclassified|f__Alphaproteobacteria_unclassified**	2|1224|28211||	1.85506
**k__Bacteria|p__Firmicutes|c__Bacilli|o__Bacillales|f__Bacillaceae**	2|1239|91061|1385|186817	1.3679
**k__Bacteria|p__Proteobacteria|c__Betaproteobacteria|o__Burkholderiales|f__Burkholderiaceae**	2|1224|28216|80840|119060	0.73303
**k__Bacteria|p__Proteobacteria|c__Gammaproteobacteria|o__Pseudomonadales|f__Perlucidibacaceae**	2|1224|1236|72274|2887331	0.69693
**k__Bacteria|p__Actinobacteria|c__Actinomycetia|o__Candidatus_Nanopelagicales|f__Candidatus_Nanopelagicaceae**	2|201174|1760|2039638|2162846	0.48415
**k__Bacteria|p__Firmicutes|c__Bacilli|o__Bacillales|f__Staphylococcaceae**	2|1239|91061|1385|90964	0.18891
**k__Bacteria|p__Actinobacteria|c__CFGB13153|o__OFGB13153|f__FGB13153**	2|201174|||	0.13493
**k__Bacteria|p__Actinobacteria|c__CFGB13659|o__OFGB13659|f__FGB13659**	2|201174|||	0.07571

**k__Bacteria|p__Proteobacteria|c__Betaproteobacteria|o__Rhodocyclales|f__Zoogloeaceae**	2|1224|28216|206389|2008794	0.05707
**k__Bacteria|p__Proteobacteria|c__CFGB76227|o__OFGB76227|f__FGB76227**	2|1224|||	0.04863
**k__Bacteria|p__Bacteroidetes|c__CFGB14294|o__OFGB14294|f__FGB14294**	2|976|||	0.04295
**k__Bacteria|p__Acidobacteria|c__CFGB13989|o__OFGB13989|f__FGB13989|g__GGB74065**	2|57723||||	23.00169
**k__Bacteria|p__Proteobacteria|c__Gammaproteobacteria|o__Moraxellales|f__Moraxellaceae|g__Acinetobacter**	2|1224|1236|2887326|468|469	19.55144
**k__Bacteria|p__Proteobacteria|c__Alphaproteobacteria|o__Pelagibacterales|f__Pelagibacterales_unclassified|g__Candidatus_Fonsibacter**	2|1224|28211|54526||2045213	17.07819
**k__Bacteria|p__Proteobacteria|c__Betaproteobacteria|o__Nitrosomonadales|f__Methylophilaceae|g__Candidatus_Methylopumilus**	2|1224|28216|32003|32011|1679002	9.49904
**k__Bacteria|p__Actinobacteria|c__CFGB34754|o__OFGB34754|f__FGB34754|g__GGB34754**	2|201174||||	7.09333
**k__Bacteria|p__Bacteroidetes|c__CFGB18754|o__OFGB18754|f__FGB18754|g__GGB46527**	2|976||||	6.58054
**k__Bacteria|p__Proteobacteria|c__Gammaproteobacteria|o__Pseudomonadales|f__Pseudomonadaceae|g__Pseudomonas**	2|1224|1236|72274|135621|286	5.34458
**k__Bacteria|p__Actinobacteria|c__Actinomycetia|o__Actinomycetia_unclassified|f__Actinomycetia_unclassified|g__Actinomycetia_unclassified**	2|201174|1760|||	3.65623
**k__Bacteria|p__Actinobacteria|c__CFGB1083|o__OFGB1083|f__FGB1083|g__GGB24856**	2|201174||||	2.29237
**k__Bacteria|p__Proteobacteria|c__Alphaproteobacteria|o__Alphaproteobacteria_unclassified|f__Alphaproteobacteria_unclassified|g__Alphaproteobacteria_unclassified**	2|1224|28211|||	1.85506
**k__Bacteria|p__Firmicutes|c__Bacilli|o__Bacillales|f__Bacillaceae|g__Bacillus**	2|1239|91061|1385|186817|1386	1.3679
**k__Bacteria|p__Proteobacteria|c__Betaproteobacteria|o__Burkholderiales|f__Burkholderiaceae|g__GGB26028**	2|1224|28216|80840|119060|	0.73303
**k__Bacteria|p__Proteobacteria|c__Gammaproteobacteria|o__Pseudomonadales|f__Perlucidibacaceae|g__Perlucidibaca**	2|1224|1236|72274|2887331|661182	0.69693
**k__Bacteria|p__Actinobacteria|c__Actinomycetia|o__Candidatus_Nanopelagicales|f__Candidatus_Nanopelagicaceae|g__Candidatus_Planktophila**	2|201174|1760|2039638|2162846|622681	0.27618
**k__Bacteria|p__Actinobacteria|c__CFGB1083|o__OFGB1083|f__FGB1083|g__GGB36422**	2|201174||||	0.21733
**k__Bacteria|p__Actinobacteria|c__Actinomycetia|o__Candidatus_Nanopelagicales|f__Candidatus_Nanopelagicaceae|g__Candidatus_Nanopelagicus**	2|201174|1760|2039638|2162846|2039639	0.20797
**k__Bacteria|p__Firmicutes|c__Bacilli|o__Bacillales|f__Staphylococcaceae|g__Staphylococcus**	2|1239|91061|1385|90964|1279	0.18891
**k__Bacteria|p__Actinobacteria|c__CFGB13153|o__OFGB13153|f__FGB13153|g__GGB38373**	2|201174||||	0.13493

<table>
<tr><td>k__Bacteria|p__Actinobacteria|c__CFGB13659|o__OFGB13659|f__FGB13659|g__GGB23686</td><td>2|201174||||</td><td>0.07571</td></tr>
<tr><td>k__Bacteria|p__Proteobacteria|c__Betaproteobacteria|o__Rhodocyclales|f__Zoogloeaceae|g__Zoogloea</td><td>2|1224|28216|206389|2008794|349</td><td>0.05707</td></tr>
<tr><td>k__Bacteria|p__Proteobacteria|c__CFGB76227|o__OFGB76227|f__FGB76227|g__GGB79622</td><td>2|1224||||</td><td>0.04863</td></tr>
<tr><td>k__Bacteria|p__Bacteroidetes|c__CFGB14294|o__OFGB14294|f__FGB14294|g__GGB26951</td><td>2|976||||</td><td>0.04295</td></tr>
<tr><td>k__Bacteria|p__Acidobacteria|c__CFGB13989|o__OFGB13989|f__FGB13989|g__GGB74065|s__GGB74065_SGB52643</td><td>2|57723|||||</td><td>23.00169</td></tr>
<tr><td>k__Bacteria|p__Proteobacteria|c__Gammaproteobacteria|o__Moraxellales|f__Moraxellaceae|g__Acinetobacter|s__Acinetobacter_bohemicus</td><td>2|1224|1236|2887326|468|469|1435036</td><td>19.55144</td></tr>
<tr><td>k__Bacteria|p__Proteobacteria|c__Alphaproteobacteria|o__Pelagibacterales|f__Pelagibacterales_unclassified|g__Candidatus_Fonsibacter|s__Candidatus_Fonsibacter_ubiquis</td><td>2|1224|28211|54526||2045213|1925548</td><td>17.07819</td></tr>
<tr><td>k__Bacteria|p__Actinobacteria|c__CFGB34754|o__OFGB34754|f__FGB34754|g__GGB34754|s__GGB34754_SGB82226</td><td>2|201174|||||</td><td>7.09333</td></tr>
<tr><td>k__Bacteria|p__Bacteroidetes|c__CFGB18754|o__OFGB18754|f__FGB18754|g__GGB46527|s__GGB46527_SGB64388</td><td>2|976|||||</td><td>6.58054</td></tr>
<tr><td>k__Bacteria|p__Proteobacteria|c__Betaproteobacteria|o__Nitrosomonadales|f__Methylophilaceae|g__Candidatus_Methylopumilus|s__Candidatus_Methylopumilus_rimovensis</td><td>2|1224|28216|32003|32011|1679002|2588535</td><td>5.47032</td></tr>
<tr><td>k__Bacteria|p__Proteobacteria|c__Betaproteobacteria|o__Nitrosomonadales|f__Methylophilaceae|g__Candidatus_Methylopumilus|s__Candidatus_Methylopumilus_universalis</td><td>2|1224|28216|32003|32011|1679002|2588536</td><td>4.02872</td></tr>
<tr><td>k__Bacteria|p__Proteobacteria|c__Gammaproteobacteria|o__Pseudomonadales|f__Pseudomonadaceae|g__Pseudomonas|s__Pseudomonas_sp_PDM05</td><td>2|1224|1236|72274|135621|286|2769301</td><td>3.5593</td></tr>
<tr><td>k__Bacteria|p__Actinobacteria|c__Actinomycetia|o__Actinomycetia_unclassified|f__Actinomycetia_unclassified|g__Actinomycetia_unclassified|s__Actinomycetia_unclassified_SGB64102</td><td>2|201174|1760||||</td><td>3.37254</td></tr>
<tr><td>k__Bacteria|p__Actinobacteria|c__CFGB1083|o__OFGB1083|f__FGB1083|g__GGB24856|s__GGB24856_SGB81948</td><td>2|201174|||||</td><td>2.29237</td></tr>
<tr><td>k__Bacteria|p__Proteobacteria|c__Alphaproteobacteria|o__Alphaproteobacteria_unclassified|f__Alphaproteobacteria_unclassified|g__Alphaproteobacteria_unclassified|s__alpha_proteobacterium_SCGC_AAA028_D10</td><td>2|1224|28211||||938641</td><td>1.85506</td></tr>
<tr><td>k__Bacteria|p__Firmicutes|c__Bacilli|o__Bacillales|f__Bacillaceae|g__Bacillus|s__Bacillus_cereus</td><td>2|1239|91061|1385|186817|1386|1396</td><td>1.3679</td></tr>
<tr><td>k__Bacteria|p__Proteobacteria|c__Gammaproteobacteria|o__Pseudomonadales|f__Pseudomonadaceae|g__Pseudomonas|s__Pseudomonas_sp_CBZ_4</td><td>2|1224|1236|72274|135621|286|1163065</td><td>0.73594</td></tr>
<tr><td>k__Bacteria|p__Proteobacteria|c__Gammaproteobacteria|o__Pseudomonadales|f__Perlucidibacaceae|g__Perlucidibaca|s__Perlucidibaca_aquatica</td><td>2|1224|1236|72274|2887331|661182|1852776</td><td>0.69693</td></tr>
<tr><td>k__Bacteria|p__Proteobacteria|c__Betaproteobacteria|o__Burkholderiales|f__Burkholderiaceae|g__GGB26028|s__GGB26028_SGB38031</td><td>2|1224|28216|80840|119060||</td><td>0.6336</td></tr>
</table>

**k__Bacteria|p__Actinobacteria|c__Actinomycetia|o__Actinomycetia_unclassified|f__Actinomycetia_unclassified|g__Actinomycetia_unclassified|s__actinobacterium_SCGC_AAA028_A23**	2|201174|1760||||932036	0.28369
**k__Bacteria|p__Proteobacteria|c__Gammaproteobacteria|o__Pseudomonadales|f__Pseudomonadaceae|g__Pseudomonas|s__Pseudomonas_viridiflava**	2|1224|1236|72274|135621|286|33069	0.27436
**k__Bacteria|p__Proteobacteria|c__Gammaproteobacteria|o__Pseudomonadales|f__Pseudomonadaceae|g__Pseudomonas|s__Pseudomonas_kielensis**	2|1224|1236|72274|135621|286|2762577	0.27416
**k__Bacteria|p__Proteobacteria|c__Gammaproteobacteria|o__Pseudomonadales|f__Pseudomonadaceae|g__Pseudomonas|s__Pseudomonas_fluorescens**	2|1224|1236|72274|135621|286|294	0.2366
**k__Bacteria|p__Actinobacteria|c__CFGB1083|o__OFGB1083|f__FGB1083|g__GGB36422|s__GGB36422_SGB61312**	2|201174|||||	0.21733
**k__Bacteria|p__Firmicutes|c__Bacilli|o__Bacillales|f__Staphylococcaceae|g__Staphylococcus|s__Staphylococcus_auricularis**	2|1239|91061|1385|90964|1279|29379	0.18891
**k__Bacteria|p__Actinobacteria|c__Actinomycetia|o__Candidatus_Nanopelagicales|f__Candidatus_Nanopelagicaceae|g__Candidatus_Nanopelagicus|s__Candidatus_Nanopelagicus_limnes**	2|201174|1760|2039638|2162846|2039639|1884634	0.17583
**k__Bacteria|p__Proteobacteria|c__Gammaproteobacteria|o__Pseudomonadales|f__Pseudomonadaceae|g__Pseudomonas|s__Pseudomonas_laurylsulfatiphila**	2|1224|1236|72274|135621|286|2011015	0.14408
**k__Bacteria|p__Actinobacteria|c__Actinomycetia|o__Candidatus_Nanopelagicales|f__Candidatus_Nanopelagicaceae|g__Candidatus_Planktophila|s__Candidatus_Planktophila_lacus**	2|201174|1760|2039638|2162846|622681|1884913	0.14236
**k__Bacteria|p__Actinobacteria|c__CFGB13153|o__OFGB13153|f__FGB13153|g__GGB38373|s__GGB38373_SGB84539**	2|201174|||||	0.13493
**k__Bacteria|p__Actinobacteria|c__Actinomycetia|o__Candidatus_Nanopelagicales|f__Candidatus_Nanopelagicaceae|g__Candidatus_Planktophila|s__Candidatus_Planktophila_vernalis**	2|201174|1760|2039638|2162846|622681|1884907	0.13382
**k__Bacteria|p__Proteobacteria|c__Betaproteobacteria|o__Burkholderiales|f__Burkholderiaceae|g__GGB26028|s__GGB26028_SGB38032**	2|1224|28216|80840|119060||	0.09943
**k__Bacteria|p__Proteobacteria|c__Gammaproteobacteria|o__Pseudomonadales|f__Pseudomonadaceae|g__Pseudomonas|s__Pseudomonas_sp_8AS**	2|1224|1236|72274|135621|286|2653163	0.08246
**k__Bacteria|p__Actinobacteria|c__CFGB13659|o__OFGB13659|f__FGB13659|g__GGB23686|s__GGB23686_SGB82209**	2|201174|||||	0.07571
**k__Bacteria|p__Proteobacteria|c__Betaproteobacteria|o__Rhodocyclales|f__Zoogloeaceae|g__Zoogloea|s__Zoogloea_oleivorans**	2|1224|28216|206389|2008794|349|1552750	0.05707
**k__Bacteria|p__Proteobacteria|c__CFGB76227|o__OFGB76227|f__FGB76227|g__GGB79622|s__GGB79622_SGB56399**	2|1224|||||	0.04863
**k__Bacteria|p__Bacteroidetes|c__CFGB14294|o__OFGB14294|f__FGB14294|g__GGB26951|s__GGB26951_SGB39152**	2|976|||||	0.04295

**k__Bacteria|p__Actinobacteria|c__Actinomycetia|o__Candidatus_Nanopelagicales|f__Candidatus_Nanopelagicaceae|g__Candidatus_Nanopelagicus|s__Candidatus_Nanopelagicus_abundans**	2|201174|1760|2039638|2162846|2039639|1884916	0.03214
**k__Bacteria|p__Proteobacteria|c__Gammaproteobacteria|o__Pseudomonadales|f__Pseudomonadaceae|g__Pseudomonas|s__Pseudomonas_sp_FW300_N2A2**	2|1224|1236|72274|135621|286|2751316	0.02014
**k__Bacteria|p__Proteobacteria|c__Gammaproteobacteria|o__Pseudomonadales|f__Pseudomonadaceae|g__Pseudomonas|s__Pseudomonas_umsongensis**	2|1224|1236|72274|135621|286|198618	0.0133
**k__Bacteria|p__Proteobacteria|c__Gammaproteobacteria|o__Pseudomonadales|f__Pseudomonadaceae|g__Pseudomonas|s__Pseudomonas_migulae**	2|1224|1236|72274|135621|286|78543	0.00424
**k__Bacteria|p__Acidobacteria|c__CFGB13989|o__OFGB13989|f__FGB13989|g__GGB74065|s__GGB74065_SGB52643|t__SGB52643**	2|57723|||||	23.00169
**k__Bacteria|p__Proteobacteria|c__Gammaproteobacteria|o__Moraxellales|f__Moraxellaceae|g__Acinetobacter|s__Acinetobacter_bohemicus|t__SGB10392**	2|1224|1236|2887326|468|469|1435036|	19.55144
**k__Bacteria|p__Proteobacteria|c__Alphaproteobacteria|o__Pelagibacterales|f__Pelagibacterales_unclassified|g__Candidatus_Fonsibacter|s__Candidatus_Fonsibacter_ubiquis|t__SGB28829**	2|1224|28211|54526||2045213|1925548|	17.07819
**k__Bacteria|p__Actinobacteria|c__CFGB34754|o__OFGB34754|f__FGB34754|g__GGB34754|s__GGB34754_SGB82226|t__SGB82226**	2|201174|||||	7.09333
**k__Bacteria|p__Bacteroidetes|c__CFGB18754|o__OFGB18754|f__FGB18754|g__GGB46527|s__GGB46527_SGB64388|t__SGB64388**	2|976|||||	6.58054
**k__Bacteria|p__Proteobacteria|c__Betaproteobacteria|o__Nitrosomonadales|f__Methylophilaceae|g__Candidatus_Methylopumilus|s__Candidatus_Methylopumilus_rimovensis|t__SGB80878**	2|1224|28216|32003|32011|1679002|2588535|	5.47032
**k__Bacteria|p__Proteobacteria|c__Betaproteobacteria|o__Nitrosomonadales|f__Methylophilaceae|g__Candidatus_Methylopumilus|s__Candidatus_Methylopumilus_universalis|t__SGB38034**	2|1224|28216|32003|32011|1679002|2588536|	4.02872
**k__Bacteria|p__Proteobacteria|c__Gammaproteobacteria|o__Pseudomonadales|f__Pseudomonadaceae|g__Pseudomonas|s__Pseudomonas_sp_PDM05|t__SGB89067**	2|1224|1236|72274|135621|286|2769301|	3.5593
**k__Bacteria|p__Actinobacteria|c__Actinomycetia|o__Actinomycetia_unclassified|f__Actinomycetia_unclassified|g__Actinomycetia_unclassified|s__Actinomycetia_unclassified_SGB64102|t__SGB64102**	2|201174|1760||||	3.37254
**k__Bacteria|p__Actinobacteria|c__CFGB1083|o__OFGB1083|f__FGB1083|g__GGB24856|s__GGB24856_SGB81948|t__SGB81948**	2|201174|||||	2.29237
**k__Bacteria|p__Proteobacteria|c__Alphaproteobacteria|o__Alphaproteobacteria_unclassified|f__Alphaproteobacteria_unclassified|g__Alphaproteobacteria_unclassified|s__alpha_proteobacterium_SCGC_AAA028_D10|t__SGB50895_group**	2|1224|28211||||938641|	1.85506
**k__Bacteria|p__Firmicutes|c__Bacilli|o__Bacillales|f__Bacillaceae|g__Bacillus|s__Bacillus_cereus|t__SGB7697**	2|1239|91061|1385|186817|1386|1396|	1.3679
**k__Bacteria|p__Proteobacteria|c__Gammaproteobacteria|o__Pseudomonadales|f__Pseudomonadaceae|g__Pseudomonas|s__Pseudomonas_sp_CBZ_4|t__SGB33231**	2|1224|1236|72274|135621|286|1163065|	0.73594

**k__Bacteria|p__Proteobacteria|c__Gammaproteobacteria|o__Pseudomonadales|f__Perlucidibacaceae|g__Perlucidibaca|s__Perlucidibaca_aquatica|t__SGB33026**	2|1224|1236|72274|2887331|661182|1852776|	0.69693
**k__Bacteria|p__Proteobacteria|c__Betaproteobacteria|o__Burkholderiales|f__Burkholderiaceae|g__GGB26028|s__GGB26028_SGB38031|t__SGB38031**	2|1224|28216|80840|119060|||	0.6336
**k__Bacteria|p__Actinobacteria|c__Actinomycetia|o__Actinomycetia_unclassified|f__Actinomycetia_unclassified|g__Actinomycetia_unclassified|s__actinobacterium_SCGC_AAA028_A23|t__SGB5713**	2|201174|1760|||932036|	0.28369
**k__Bacteria|p__Proteobacteria|c__Gammaproteobacteria|o__Pseudomonadales|f__Pseudomonadaceae|g__Pseudomonas|s__Pseudomonas_viridiflava|t__SGB12132**	2|1224|1236|72274|135621|286|33069|	0.27436
**k__Bacteria|p__Proteobacteria|c__Gammaproteobacteria|o__Pseudomonadales|f__Pseudomonadaceae|g__Pseudomonas|s__Pseudomonas_kielensis|t__SGB89079**	2|1224|1236|72274|135621|286|2762577|	0.27416
**k__Bacteria|p__Proteobacteria|c__Gammaproteobacteria|o__Pseudomonadales|f__Pseudomonadaceae|g__Pseudomonas|s__Pseudomonas_fluorescens|t__SGB12163**	2|1224|1236|72274|135621|286|294|	0.2366
**k__Bacteria|p__Actinobacteria|c__CFGB1083|o__OFGB1083|f__FGB1083|g__GGB36422|s__GGB36422_SGB61312|t__SGB61312**	2|201174|||||	0.21733
**k__Bacteria|p__Firmicutes|c__Bacilli|o__Bacillales|f__Staphylococcaceae|g__Staphylococcus|s__Staphylococcus_auricularis|t__SGB7816**	2|1239|91061|1385|90964|1279|29379|	0.18891
**k__Bacteria|p__Actinobacteria|c__Actinomycetia|o__Candidatus_Nanopelagicales|f__Candidatus_Nanopelagicaceae|g__Candidatus_Nanopelagicus|s__Candidatus_Nanopelagicus_limnes|t__SGB5711**	2|201174|1760|2039638|2162846|2039639|1884634|	0.17583
**k__Bacteria|p__Proteobacteria|c__Gammaproteobacteria|o__Pseudomonadales|f__Pseudomonadaceae|g__Pseudomonas|s__Pseudomonas_laurylsulfatiphila|t__SGB12221**	2|1224|1236|72274|135621|286|2011015|	0.14408
**k__Bacteria|p__Actinobacteria|c__Actinomycetia|o__Candidatus_Nanopelagicales|f__Candidatus_Nanopelagicaceae|g__Candidatus_Planktophila|s__Candidatus_Planktophila_lacus|t__SGB5706**	2|201174|1760|2039638|2162846|622681|1884913|	0.14236
**k__Bacteria|p__Actinobacteria|c__CFGB13153|o__OFGB13153|f__FGB13153|g__GGB38373|s__GGB38373_SGB84539|t__SGB84539**	2|201174|||||	0.13493
**k__Bacteria|p__Actinobacteria|c__Actinomycetia|o__Candidatus_Nanopelagicales|f__Candidatus_Nanopelagicaceae|g__Candidatus_Planktophila|s__Candidatus_Planktophila_vernalis|t__SGB5705**	2|201174|1760|2039638|2162846|622681|1884907|	0.13382
**k__Bacteria|p__Proteobacteria|c__Betaproteobacteria|o__Burkholderiales|f__Burkholderiaceae|g__GGB26028|s__GGB26028_SGB38032|t__SGB38032**	2|1224|28216|80840|119060|||	0.09943
**k__Bacteria|p__Proteobacteria|c__Gammaproteobacteria|o__Pseudomonadales|f__Pseudomonadaceae|g__Pseudomonas|s__Pseudomonas_sp_8AS|t__SGB89176**	2|1224|1236|72274|135621|286|2653163|	0.08246
**k__Bacteria|p__Actinobacteria|c__CFGB13659|o__OFGB13659|f__FGB13659|g__GGB23686|s__GGB23686_SGB82209|t__SGB82209**	2|201174|||||	0.07571
**k__Bacteria|p__Proteobacteria|c__Betaproteobacteria|o__Rhodocyclales|f__Zoogloeaceae|g__Zoogloea|s__Zoogloea_oleivorans|t__SGB88263**	2|1224|28216|206389|2008794|349|1552750|	0.05707

**k__Bacteria|p__Proteobacteria|c__CFGB76227|o__OFGB76227|f__FGB76227|g__GGB79622|s__GGB79622_SGB56399|t__SGB56399**	2|1224|||||	0.04863
**k__Bacteria|p__Bacteroidetes|c__CFGB14294|o__OFGB14294|f__FGB14294|g__GGB26951|s__GGB26951_SGB39152|t__SGB39152**	2|976|||||	0.04295
**k__Bacteria|p__Actinobacteria|c__Actinomycetia|o__Candidatus_Nanopelagicales|f__Candidatus_Nanopelagicaceae|g__Candidatus_Nanopelagicus|s__Candidatus_Nanopelagicus_abundans|t__SGB5714**	2|201174|1760|2039638|2162846|2039639|1884916|	0.03214
**k__Bacteria|p__Proteobacteria|c__Gammaproteobacteria|o__Pseudomonadales|f__Pseudomonadaceae|g__Pseudomonas|s__Pseudomonas_sp_FW300_N2A2|t__SGB12242**	2|1224|1236|72274|135621|286|2751316|	0.02014
**k__Bacteria|p__Proteobacteria|c__Gammaproteobacteria|o__Pseudomonadales|f__Pseudomonadaceae|g__Pseudomonas|s__Pseudomonas_umsongensis|t__SGB12217**	2|1224|1236|72274|135621|286|198618|	0.0133
**k__Bacteria|p__Proteobacteria|c__Gammaproteobacteria|o__Pseudomonadales|f__Pseudomonadaceae|g__Pseudomonas|s__Pseudomonas_migulae|t__SGB12244**	2|1224|1236|72274|135621|286|78543|	0.00424

Table S4. Relative abundance of Bacteria found in the Welbedacht Raw Summer samples generated from MetaPhlan used to create Figure 1.

#SampleID	Metaphlan_Analysis	
#clade_name	NCBI_tax_id	relative_abundance
**k__Bacteria**	2	100.0
**k__Bacteria|p__Proteobacteria**	2|1224	76.50115
**k__Bacteria|p__Planctomycetes**	2|203682	11.60069
**k__Bacteria|p__Bacteroidetes**	2|976	7.53031
**k__Bacteria|p__Actinobacteria**	2|201174	2.30474
**k__Bacteria|p__Candidatus_Kryptonia**	2|1855361	2.01622
**k__Bacteria|p__Firmicutes**	2|1239	0.04689
**k__Bacteria|p__Proteobacteria|c__Alphaproteobacteria**	2|1224|28211	36.13726
**k__Bacteria|p__Proteobacteria|c__Betaproteobacteria**	2|1224|28216	20.41083
**k__Bacteria|p__Proteobacteria|c__Gammaproteobacteria**	2|1224|1236	19.95306
**k__Bacteria|p__Planctomycetes|c__CFGB8429**	2|203682|	11.60069
**k__Bacteria|p__Bacteroidetes|c__CFGB18754**	2|976|	4.27775
**k__Bacteria|p__Bacteroidetes|c__CFGB40560**	2|976|	3.23647
**k__Bacteria|p__Candidatus_Kryptonia|c__CFGB41117**	2|1855361|	2.01622
**k__Bacteria|p__Actinobacteria|c__Actinomycetia**	2|201174|1760	1.86853

**k__Bacteria|p__Actinobacteria|c__CFGB46334**	2|201174|	0.43621
**k__Bacteria|p__Firmicutes|c__Bacilli**	2|1239|91061	0.04689
**k__Bacteria|p__Bacteroidetes|c__Sphingobacteriia**	2|976|117747	0.01608
**k__Bacteria|p__Proteobacteria|c__Betaproteobacteria|o__Nitrosomonadales**	2|1224|28216|32003	20.30023
**k__Bacteria|p__Proteobacteria|c__Gammaproteobacteria|o__Pseudomonadales**	2|1224|1236|72274	19.95306
**k__Bacteria|p__Proteobacteria|c__Alphaproteobacteria|o__Pelagibacterales**	2|1224|28211|54526	19.24233
**k__Bacteria|p__Proteobacteria|c__Alphaproteobacteria|o__Alphaproteobacteria_unclassified**	2|1224|28211|	16.89493
**k__Bacteria|p__Planctomycetes|c__CFGB8429|o__OFGB8429**	2|203682||	11.60069
**k__Bacteria|p__Bacteroidetes|c__CFGB18754|o__OFGB18754**	2|976||	4.27775
**k__Bacteria|p__Bacteroidetes|c__CFGB40560|o__OFGB40560**	2|976||	3.23647
**k__Bacteria|p__Candidatus_Kryptonia|c__CFGB41117|o__OFGB41117**	2|1855361||	2.01622
**k__Bacteria|p__Actinobacteria|c__Actinomycetia|o__Candidatus_Nanopelagicales**	2|201174|1760|2039638	1.39272
**k__Bacteria|p__Actinobacteria|c__CFGB46334|o__OFGB46334**	2|201174||	0.43621
**k__Bacteria|p__Actinobacteria|c__Actinomycetia|o__Actinomycetia_unclassified**	2|201174|1760|	0.31351
**k__Bacteria|p__Actinobacteria|c__Actinomycetia|o__Bifidobacteriales**	2|201174|1760|85004	0.1623
**k__Bacteria|p__Proteobacteria|c__Betaproteobacteria|o__Burkholderiales**	2|1224|28216|80840	0.1106
**k__Bacteria|p__Firmicutes|c__Bacilli|o__Bacillales**	2|1239|91061|1385	0.04689
**k__Bacteria|p__Bacteroidetes|c__Sphingobacteriia|o__Sphingobacteriales**	2|976|117747|200666	0.01608
**k__Bacteria|p__Proteobacteria|c__Betaproteobacteria|o__Nitrosomonadales|f__Methylophilaceae**	2|1224|28216|32003|32011	20.01485
**k__Bacteria|p__Proteobacteria|c__Gammaproteobacteria|o__Pseudomonadales|f__Pseudomonadaceae**	2|1224|1236|72274|135621	19.95306
**k__Bacteria|p__Proteobacteria|c__Alphaproteobacteria|o__Pelagibacterales|f__Pelagibacterales_unclassified**	2|1224|28211|54526|	19.24233
**k__Bacteria|p__Proteobacteria|c__Alphaproteobacteria|o__Alphaproteobacteria_unclassified|f__Alphaproteobacteria_unclassified**	2|1224|28211||	16.89493
**k__Bacteria|p__Planctomycetes|c__CFGB8429|o__OFGB8429|f__FGB8429**	2|203682|||	11.60069
**k__Bacteria|p__Bacteroidetes|c__CFGB18754|o__OFGB18754|f__FGB18754**	2|976|||	4.27775
**k__Bacteria|p__Bacteroidetes|c__CFGB40560|o__OFGB40560|f__FGB40560**	2|976|||	3.23647
**k__Bacteria|p__Candidatus_Kryptonia|c__CFGB41117|o__OFGB41117|f__FGB41117**	2|1855361|||	2.01622
**k__Bacteria|p__Actinobacteria|c__Actinomycetia|o__Candidatus_Nanopelagicales|f__Candidatus_Nanopelagicaceae**	2|201174|1760|2039638|2162846	1.39272

**k__Bacteria|p__Actinobacteria|c__CFGB46334|o__OFGB46334|f__FGB46334**	2|201174|||	0.43621
**k__Bacteria|p__Actinobacteria|c__Actinomycetia|o__Actinomycetia_unclassified|f__Actinomycetia_unclassified**	2|201174|1760||	0.31351
**k__Bacteria|p__Proteobacteria|c__Betaproteobacteria|o__Nitrosomonadales|f__Nitrosomonadaceae**	2|1224|28216|32003|206379	0.28537
**k__Bacteria|p__Actinobacteria|c__Actinomycetia|o__Bifidobacteriales|f__Bifidobacteriaceae**	2|201174|1760|85004|31953	0.1623
**k__Bacteria|p__Proteobacteria|c__Betaproteobacteria|o__Burkholderiales|f__Burkholderiaceae**	2|1224|28216|80840|119060	0.1106
**k__Bacteria|p__Firmicutes|c__Bacilli|o__Bacillales|f__Staphylococcaceae**	2|1239|91061|1385|90964	0.04689
**k__Bacteria|p__Bacteroidetes|c__Sphingobacteriia|o__Sphingobacteriales|f__Sphingobacteriaceae**	2|976|117747|200666|84566	0.01608
**k__Bacteria|p__Proteobacteria|c__Betaproteobacteria|o__Nitrosomonadales|f__Methylophilaceae|g__Candidatus_Methylopumilus**	2|1224|28216|32003|32011|1679002	20.01485
**k__Bacteria|p__Proteobacteria|c__Gammaproteobacteria|o__Pseudomonadales|f__Pseudomonadaceae|g__Pseudomonas**	2|1224|1236|72274|135621|286	19.95306
**k__Bacteria|p__Proteobacteria|c__Alphaproteobacteria|o__Pelagibacterales|f__Pelagibacterales_unclassified|g__Candidatus_Fonsibacter**	2|1224|28211|54526||2045213	19.24233
**k__Bacteria|p__Proteobacteria|c__Alphaproteobacteria|o__Alphaproteobacteria_unclassified|f__Alphaproteobacteria_unclassified|g__Alphaproteobacteria_unclassified**	2|1224|28211|||	16.89493
**k__Bacteria|p__Planctomycetes|c__CFGB8429|o__OFGB8429|f__FGB8429|g__GGB25723**	2|203682||||	11.60069
**k__Bacteria|p__Bacteroidetes|c__CFGB18754|o__OFGB18754|f__FGB18754|g__GGB46527**	2|976||||	4.27775
**k__Bacteria|p__Bacteroidetes|c__CFGB40560|o__OFGB40560|f__FGB40560|g__GGB43022**	2|976||||	3.23647
**k__Bacteria|p__Candidatus_Kryptonia|c__CFGB41117|o__OFGB41117|f__FGB41117|g__GGB75394**	2|1855361||||	2.01622
**k__Bacteria|p__Actinobacteria|c__Actinomycetia|o__Candidatus_Nanopelagicales|f__Candidatus_Nanopelagicaceae|g__Candidatus_Nanopelagicus**	2|201174|1760|2039638|2162846|2039639	1.11644
**k__Bacteria|p__Actinobacteria|c__CFGB46334|o__OFGB46334|f__FGB46334|g__GGB46334**	2|201174||||	0.43621
**k__Bacteria|p__Actinobacteria|c__Actinomycetia|o__Actinomycetia_unclassified|f__Actinomycetia_unclassified|g__Actinomycetia_unclassified**	2|201174|1760|||	0.31351
**k__Bacteria|p__Proteobacteria|c__Betaproteobacteria|o__Nitrosomonadales|f__Nitrosomonadaceae|g__Nitrosospira**	2|1224|28216|32003|206379|35798	0.28537
**k__Bacteria|p__Actinobacteria|c__Actinomycetia|o__Candidatus_Nanopelagicales|f__Candidatus_Nanopelagicaceae|g__Candidatus_Planktophila**	2|201174|1760|2039638|2162846|622681	0.27628

**k__Bacteria|p__Actinobacteria|c__Actinomycetia|o__Bifidobacteriales|f__Bifidobacteriaceae|g__Gardnerella**	2|201174|1760|85004|31953|2701	0.1623
**k__Bacteria|p__Proteobacteria|c__Betaproteobacteria|o__Burkholderiales|f__Burkholderiaceae|g__GGB26028**	2|1224|28216|80840|119060|	0.1106
**k__Bacteria|p__Firmicutes|c__Bacilli|o__Bacillales|f__Staphylococcaceae|g__Staphylococcus**	2|1239|91061|1385|90964|1279	0.04689
**k__Bacteria|p__Bacteroidetes|c__Sphingobacteriia|o__Sphingobacteriales|f__Sphingobacteriaceae|g__Daejeonella**	2|976|117747|200666|84566|2762385	0.01608
**k__Bacteria|p__Proteobacteria|c__Gammaproteobacteria|o__Pseudomonadales|f__Pseudomonadaceae|g__Pseudomonas|s__Pseudomonas_sp_PDM05**	2|1224|1236|72274|135621|286|2769301	19.95306
**k__Bacteria|p__Proteobacteria|c__Betaproteobacteria|o__Nitrosomonadales|f__Methylophilaceae|g__Candidatus_Methylopumilus|s__Candidatus_Methylopumilus_universalis**	2|1224|28216|32003|32011|1679002|2588536	19.74126
**k__Bacteria|p__Proteobacteria|c__Alphaproteobacteria|o__Pelagibacterales|f__Pelagibacterales_unclassified|g__Candidatus_Fonsibacter|s__Candidatus_Fonsibacter_ubiquis**	2|1224|28211|54526||2045213|1925548	19.24233
**k__Bacteria|p__Proteobacteria|c__Alphaproteobacteria|o__Alphaproteobacteria_unclassified|f__Alphaproteobacteria_unclassified|g__Alphaproteobacteria_unclassified|s__alpha_proteobacterium_SCGC_AAA028_D10**	2|1224|28211||||938641	16.89493
**k__Bacteria|p__Planctomycetes|c__CFGB8429|o__OFGB8429|f__FGB8429|g__GGB25723|s__GGB25723_SGB84803**	2|203682|||||	11.60069
**k__Bacteria|p__Bacteroidetes|c__CFGB18754|o__OFGB18754|f__FGB18754|g__GGB46527|s__GGB46527_SGB64388**	2|976|||||	4.27775
**k__Bacteria|p__Bacteroidetes|c__CFGB40560|o__OFGB40560|f__FGB40560|g__GGB43022|s__GGB43022_SGB60257**	2|976|||||	3.23647
**k__Bacteria|p__Candidatus_Kryptonia|c__CFGB41117|o__OFGB41117|f__FGB41117|g__GGB75394|s__GGB75394_SGB103389**	2|1855361|||||	2.01622
**k__Bacteria|p__Actinobacteria|c__Actinomycetia|o__Candidatus_Nanopelagicales|f__Candidatus_Nanopelagicaceae|g__Candidatus_Nanopelagicus|s__Candidatus_Nanopelagicus_limnes**	2|201174|1760|2039638|2162846|2039639|1884634	0.98878
**k__Bacteria|p__Actinobacteria|c__CFGB46334|o__OFGB46334|f__FGB46334|g__GGB46334|s__GGB46334_SGB35380**	2|201174|||||	0.43621
**k__Bacteria|p__Proteobacteria|c__Betaproteobacteria|o__Nitrosomonadales|f__Nitrosomonadaceae|g__Nitrosospira|s__Nitrosospira_sp_Nsp13**	2|1224|28216|32003|206379|35798|1855332	0.28537

**k__Bacteria|p__Proteobacteria|c__Betaproteobacteria|o__Nitrosomonadales|f__Methylophilaceae|g__Candidatus_Methylopumilus|s__Candidatus_Methylopumilus_rimovensis**	2|1224|28216|32003|32011|1679002|2588535	0.27359
**k__Bacteria|p__Actinobacteria|c__Actinomycetia|o__Actinomycetia_unclassified|f__Actinomycetia_unclassified|g__Actinomycetia_unclassified|s__actinobacterium_SCGC_AAA028_A23**	2|201174|1760||||932036	0.20112
**k__Bacteria|p__Actinobacteria|c__Actinomycetia|o__Bifidobacteriales|f__Bifidobacteriaceae|g__Gardnerella|s__Gardnerella_vaginalis**	2|201174|1760|85004|31953|2701|2702	0.1623
**k__Bacteria|p__Actinobacteria|c__Actinomycetia|o__Candidatus_Nanopelagicales|f__Candidatus_Nanopelagicaceae|g__Candidatus_Planktophila|s__Candidatus_Planktophila_vernalis**	2|201174|1760|2039638|2162846|622681|1884907	0.14786
**k__Bacteria|p__Actinobacteria|c__Actinomycetia|o__Candidatus_Nanopelagicales|f__Candidatus_Nanopelagicaceae|g__Candidatus_Planktophila|s__Candidatus_Planktophila_lacus**	2|201174|1760|2039638|2162846|622681|1884913	0.12842
**k__Bacteria|p__Actinobacteria|c__Actinomycetia|o__Candidatus_Nanopelagicales|f__Candidatus_Nanopelagicaceae|g__Candidatus_Nanopelagicus|s__Candidatus_Nanopelagicus_abundans**	2|201174|1760|2039638|2162846|2039639|1884916	0.12766
**k__Bacteria|p__Actinobacteria|c__Actinomycetia|o__Actinomycetia_unclassified|f__Actinomycetia_unclassified|g__Actinomycetia_unclassified|s__Actinomycetia_unclassified_SGB64102**	2|201174|1760||||	0.11239
**k__Bacteria|p__Proteobacteria|c__Betaproteobacteria|o__Burkholderiales|f__Burkholderiaceae|g__GGB26028|s__GGB26028_SGB38032**	2|1224|28216|80840|119060||	0.1106
**k__Bacteria|p__Firmicutes|c__Bacilli|o__Bacillales|f__Staphylococcaceae|g__Staphylococcus|s__Staphylococcus_epidermidis**	2|1239|91061|1385|90964|1279|1282	0.04689
**k__Bacteria|p__Bacteroidetes|c__Sphingobacteriia|o__Sphingobacteriales|f__Sphingobacteriaceae|g__Daejeonella|s__Daejeonella_rubra**	2|976|117747|200666|84566|2762385|990371	0.01608
**k__Bacteria|p__Proteobacteria|c__Gammaproteobacteria|o__Pseudomonadales|f__Pseudomonadaceae|g__Pseudomonas|s__Pseudomonas_sp_PDM05|t__SGB89067**	2|1224|1236|72274|135621|286|2769301|	19.95306
**k__Bacteria|p__Proteobacteria|c__Betaproteobacteria|o__Nitrosomonadales|f__Methylophilaceae|g__Candidatus_Methylopumilus|s__Candidatus_Methylopumilus_universalis|t__SGB38034**	2|1224|28216|32003|32011|1679002|2588536|	19.74126
**k__Bacteria|p__Proteobacteria|c__Alphaproteobacteria|o__Pelagibacterales|f__Pelagibacterales_unclassified|g__Candidatus_Fonsibacter|s__Candidatus_Fonsibacter_ubiquis|t__SGB28829**	2|1224|28211|54526||2045213|1925548|	19.24233
**k__Bacteria|p__Proteobacteria|c__Alphaproteobacteria|o__Alphaproteobacteria_unclassified|f__Alphaproteobacteria_unclassified|g__Alphaproteobacteria_unclassified|s__alpha_proteobacterium_SCGC_AAA028_D10|t__SGB50895_group**	2|1224|28211||||938641|	16.89493

**k__Bacteria|p__Planctomycetes|c__CFGB8429|o__OFGB8429|f__FGB8429|g__GGB25723|s__GGB25723_SGB84803|t__SGB84803**	2|203682||||||	11.60069
**k__Bacteria|p__Bacteroidetes|c__CFGB18754|o__OFGB18754|f__FGB18754|g__GGB46527|s__GGB46527_SGB64388|t__SGB64388**	2|976||||||	4.27775
**k__Bacteria|p__Bacteroidetes|c__CFGB40560|o__OFGB40560|f__FGB40560|g__GGB43022|s__GGB43022_SGB60257|t__SGB60257**	2|976||||||	3.23647
**k__Bacteria|p__Candidatus_Kryptonia|c__CFGB41117|o__OFGB41117|f__FGB41117|g__GGB75394|s__GGB75394_SGB103389|t__SGB103389**	2|1855361||||||	2.01622
**k__Bacteria|p__Actinobacteria|c__Actinomycetia|o__Candidatus_Nanopelagicales|f__Candidatus_Nanopelagicaceae|g__Candidatus_Nanopelagicus|s__Candidatus_Nanopelagicus_limnes|t__SGB5711**	2|201174|1760|2039638|2162846|2039639|1884634|	0.98878
**k__Bacteria|p__Actinobacteria|c__CFGB46334|o__OFGB46334|f__FGB46334|g__GGB46334|s__GGB46334_SGB35380|t__SGB35380**	2|201174||||||	0.43621
**k__Bacteria|p__Proteobacteria|c__Betaproteobacteria|o__Nitrosomonadales|f__Nitrosomonadaceae|g__Nitrosospira|s__Nitrosospira_sp_Nsp13|t__SGB13341**	2|1224|28216|32003|206379|35798|1855332|	0.28537
**k__Bacteria|p__Proteobacteria|c__Betaproteobacteria|o__Nitrosomonadales|f__Methylophilaceae|g__Candidatus_Methylopumilus|s__Candidatus_Methylopumilus_rimovensis|t__SGB80878**	2|1224|28216|32003|32011|1679002|2588535|	0.27359
**k__Bacteria|p__Actinobacteria|c__Actinomycetia|o__Actinomycetia_unclassified|f__Actinomycetia_unclassified|g__Actinomycetia_unclassified|s__actinobacterium_SCGC_AAA028_A23|t__SGB5713**	2|201174|1760||||932036|	0.20112
**k__Bacteria|p__Actinobacteria|c__Actinomycetia|o__Bifidobacteriales|f__Bifidobacteriaceae|g__Gardnerella|s__Gardnerella_vaginalis|t__SGB17307**	2|201174|1760|85004|31953|2701|2702|	0.1623
**k__Bacteria|p__Actinobacteria|c__Actinomycetia|o__Candidatus_Nanopelagicales|f__Candidatus_Nanopelagicaceae|g__Candidatus_Planktophila|s__Candidatus_Planktophila_vernalis|t__SGB5705**	2|201174|1760|2039638|2162846|622681|1884907|	0.14786
**k__Bacteria|p__Actinobacteria|c__Actinomycetia|o__Candidatus_Nanopelagicales|f__Candidatus_Nanopelagicaceae|g__Candidatus_Planktophila|s__Candidatus_Planktophila_lacus|t__SGB5706**	2|201174|1760|2039638|2162846|622681|1884913|	0.12842
**k__Bacteria|p__Actinobacteria|c__Actinomycetia|o__Candidatus_Nanopelagicales|f__Candidatus_Nanopelagicaceae|g__Candidatus_Nanopelagicus|s__Candidatus_Nanopelagicus_abundans|t__SGB5714**	2|201174|1760|2039638|2162846|2039639|1884916|	0.12766
**k__Bacteria|p__Actinobacteria|c__Actinomycetia|o__Actinomycetia_unclassified|f__Actinomycetia_unclassified|g__Actinomycetia_unclassified|s__Actinomycetia_unclassified_SGB64102|t__SGB64102**	2|201174|1760|||||	0.11239

**k__Bacteria|p__Proteobacteria|c__Betaproteobacteria|o__Burkholderiales|f__Burkholderiaceae|g__GGB26028|s__GGB26028_SGB38032|t__SGB38032**	2|1224|28216|80840|119060|||	0.1106
**k__Bacteria|p__Firmicutes|c__Bacilli|o__Bacillales|f__Staphylococcaceae|g__Staphylococcus|s__Staphylococcus_epidermidis|t__SGB7865**	2|1239|91061|1385|90964|1279|1282|	0.04689
**k__Bacteria|p__Bacteroidetes|c__Sphingobacteriia|o__Sphingobacteriales|f__Sphingobacteriaceae|g__Daejeonella|s__Daejeonella_rubra|t__SGB3127**	2|976|117747|200666|84566|2762385|990371|	0.01608

1.

Table S5. Relative abundance of Bacteria found in the Welbedacht Raw Winter samples generated from MetaPhlan used to create Figure 1.

#SampleID	**Metaphlan_Analysis**	
**#clade_name**	NCBI_tax_id	relative_abundance
**k__Bacteria**	2	100.0
**k__Bacteria|p__Proteobacteria**	2|1224	83.64969
**k__Bacteria|p__Candidatus_Kryptonia**	2|1855361	5.04592
**k__Bacteria|p__Planctomycetes**	2|203682	4.76934
**k__Bacteria|p__Actinobacteria**	2|201174	4.36653
**k__Bacteria|p__Bacteroidetes**	2|976	2.15006
**k__Bacteria|p__Nitrospirae**	2|40117	0.01845
**k__Bacteria|p__Proteobacteria|c__Gammaproteobacteria**	2|1224|1236	46.16564
**k__Bacteria|p__Proteobacteria|c__Alphaproteobacteria**	2|1224|28211	22.68254
**k__Bacteria|p__Proteobacteria|c__Betaproteobacteria**	2|1224|28216	14.67886
**k__Bacteria|p__Candidatus_Kryptonia|c__CFGB41117**	2|1855361|	5.04592
**k__Bacteria|p__Planctomycetes|c__CFGB8429**	2|203682|	4.76934
**k__Bacteria|p__Actinobacteria|c__Actinomycetia**	2|201174|1760	3.56941
**k__Bacteria|p__Bacteroidetes|c__CFGB40560**	2|976|	1.08541
**k__Bacteria|p__Bacteroidetes|c__CFGB18754**	2|976|	1.01439
**k__Bacteria|p__Actinobacteria|c__CFGB46334**	2|201174|	0.6312
**k__Bacteria|p__Proteobacteria|c__CFGB42731**	2|1224|	0.12265
**k__Bacteria|p__Actinobacteria|c__CFGB13659**	2|201174|	0.07377
**k__Bacteria|p__Actinobacteria|c__CFGB1083**	2|201174|	0.05903

**k__Bacteria|p__Actinobacteria|c__CFGB34754**	2|201174|	0.03313
**k__Bacteria|p__Bacteroidetes|c__CFGB41865**	2|976|	0.02749
**k__Bacteria|p__Bacteroidetes|c__Sphingobacteriia**	2|976|117747	0.02278
**k__Bacteria|p__Nitrospirae|c__Nitrospira**	2|40117|203693	0.01845
**k__Bacteria|p__Proteobacteria|c__Gammaproteobacteria|o__Pseudomonadales**	2|1224|1236|72274	46.16564
**k__Bacteria|p__Proteobacteria|c__Betaproteobacteria|o__Nitrosomonadales**	2|1224|28216|32003	14.0474
**k__Bacteria|p__Proteobacteria|c__Alphaproteobacteria|o__Alphaproteobacteria_unclassified**	2|1224|28211|	13.08046
**k__Bacteria|p__Proteobacteria|c__Alphaproteobacteria|o__Pelagibacterales**	2|1224|28211|54526	9.60208
**k__Bacteria|p__Candidatus_Kryptonia|c__CFGB41117|o__OFGB41117**	2|1855361||	5.04592
**k__Bacteria|p__Planctomycetes|c__CFGB8429|o__OFGB8429**	2|203682||	4.76934
**k__Bacteria|p__Actinobacteria|c__Actinomycetia|o__Candidatus_Nanopelagicales**	2|201174|1760|2039638	2.56456
**k__Bacteria|p__Bacteroidetes|c__CFGB40560|o__OFGB40560**	2|976||	1.08541
**k__Bacteria|p__Bacteroidetes|c__CFGB18754|o__OFGB18754**	2|976||	1.01439
**k__Bacteria|p__Actinobacteria|c__Actinomycetia|o__Actinomycetia_unclassified**	2|201174|1760|	0.99582
**k__Bacteria|p__Actinobacteria|c__CFGB46334|o__OFGB46334**	2|201174||	0.6312
**k__Bacteria|p__Proteobacteria|c__Betaproteobacteria|o__Burkholderiales**	2|1224|28216|80840	0.44443
**k__Bacteria|p__Proteobacteria|c__Betaproteobacteria|o__Betaproteobacteria_unclassified**	2|1224|28216|	0.18702
**k__Bacteria|p__Proteobacteria|c__CFGB42731|o__OFGB42731**	2|1224||	0.12265
**k__Bacteria|p__Actinobacteria|c__CFGB13659|o__OFGB13659**	2|201174||	0.07377
**k__Bacteria|p__Actinobacteria|c__CFGB1083|o__OFGB1083**	2|201174||	0.05903
**k__Bacteria|p__Actinobacteria|c__CFGB34754|o__OFGB34754**	2|201174||	0.03313
**k__Bacteria|p__Bacteroidetes|c__CFGB41865|o__OFGB41865**	2|976||	0.02749
**k__Bacteria|p__Bacteroidetes|c__Sphingobacteriia|o__Sphingobacteriales**	2|976|117747|200666	0.02278
**k__Bacteria|p__Nitrospirae|c__Nitrospira|o__Nitrospirales**	2|40117|203693|189778	0.01845
**k__Bacteria|p__Actinobacteria|c__Actinomycetia|o__Bifidobacteriales**	2|201174|1760|85004	0.00903
**k__Bacteria|p__Proteobacteria|c__Gammaproteobacteria|o__Pseudomonadales|f__Pseudomonadaceae**	2|1224|1236|72274|135621	46.16564
**k__Bacteria|p__Proteobacteria|c__Betaproteobacteria|o__Nitrosomonadales|f__Methylophilaceae**	2|1224|28216|32003|32011	13.20025
**k__Bacteria|p__Proteobacteria|c__Alphaproteobacteria|o__Alphaproteobacteria_unclassified|f__Alphaproteobacteria_unclassified**	2|1224|28211||	13.08046
**k__Bacteria|p__Proteobacteria|c__Alphaproteobacteria|o__Pelagibacterales|f__Pelagibacterales_unclassified**	2|1224|28211|54526|	9.60208

**k__Bacteria|p__Candidatus_Kryptonia|c__CFGB41117|o__OFGB41117|f__FGB41117**	2|1855361|||	5.04592
**k__Bacteria|p__Planctomycetes|c__CFGB8429|o__OFGB8429|f__FGB8429**	2|203682|||	4.76934
**k__Bacteria|p__Actinobacteria|c__Actinomycetia|o__Candidatus_Nanopelagicales|f__Candidatus_Nanopelagicaceae**	2|201174|1760|2039638|2162846	2.56456
**k__Bacteria|p__Bacteroidetes|c__CFGB40560|o__OFGB40560|f__FGB40560**	2|976|||	1.08541
**k__Bacteria|p__Bacteroidetes|c__CFGB18754|o__OFGB18754|f__FGB18754**	2|976|||	1.01439
**k__Bacteria|p__Actinobacteria|c__Actinomycetia|o__Actinomycetia_unclassified|f__Actinomycetia_unclassified**	2|201174|1760||	0.99582
**k__Bacteria|p__Proteobacteria|c__Betaproteobacteria|o__Nitrosomonadales|f__Nitrosomonadaceae**	2|1224|28216|32003|206379	0.84715
**k__Bacteria|p__Actinobacteria|c__CFGB46334|o__OFGB46334|f__FGB46334**	2|201174|||	0.6312
**k__Bacteria|p__Proteobacteria|c__Betaproteobacteria|o__Burkholderiales|f__Burkholderiaceae**	2|1224|28216|80840|119060	0.44443
**k__Bacteria|p__Proteobacteria|c__Betaproteobacteria|o__Betaproteobacteria_unclassified|f__Betaproteobacteria_unclassified**	2|1224|28216||	0.18702
**k__Bacteria|p__Proteobacteria|c__CFGB42731|o__OFGB42731|f__FGB42731**	2|1224|||	0.12265
**k__Bacteria|p__Actinobacteria|c__CFGB13659|o__OFGB13659|f__FGB13659**	2|201174|||	0.07377
**k__Bacteria|p__Actinobacteria|c__CFGB1083|o__OFGB1083|f__FGB1083**	2|201174|||	0.05903
**k__Bacteria|p__Actinobacteria|c__CFGB34754|o__OFGB34754|f__FGB34754**	2|201174|||	0.03313
**k__Bacteria|p__Bacteroidetes|c__CFGB41865|o__OFGB41865|f__FGB41865**	2|976|||	0.02749
**k__Bacteria|p__Bacteroidetes|c__Sphingobacteriia|o__Sphingobacteriales|f__Sphingobacteriaceae**	2|976|117747|200666|84566	0.02278
**k__Bacteria|p__Nitrospirae|c__Nitrospira|o__Nitrospirales|f__Nitrospiraceae**	2|40117|203693|189778|189779	0.01845
**k__Bacteria|p__Actinobacteria|c__Actinomycetia|o__Bifidobacteriales|f__Bifidobacteriaceae**	2|201174|1760|85004|31953	0.00903
**k__Bacteria|p__Proteobacteria|c__Gammaproteobacteria|o__Pseudomonadales|f__Pseudomonadaceae|g__Pseudomonas**	2|1224|1236|72274|135621|286	46.16564
**k__Bacteria|p__Proteobacteria|c__Betaproteobacteria|o__Nitrosomonadales|f__Methylophilaceae|g__Candidatus_Methylopumilus**	2|1224|28216|32003|32011|1679002	13.09233
**k__Bacteria|p__Proteobacteria|c__Alphaproteobacteria|o__Alphaproteobacteria_unclassified|f__Alphaproteobacteria_unclassified|g__Alphaproteobacteria_unclassified**	2|1224|28211|||	13.08046
**k__Bacteria|p__Proteobacteria|c__Alphaproteobacteria|o__Pelagibacterales|f__Pelagibacterales_unclassified|g__Candidatus_Fonsibacter**	2|1224|28211|54526||2045213	9.60208
**k__Bacteria|p__Candidatus_Kryptonia|c__CFGB41117|o__OFGB41117|f__FGB41117|g__GGB75394**	2|1855361||||	5.04592
**k__Bacteria|p__Planctomycetes|c__CFGB8429|o__OFGB8429|f__FGB8429|g__GGB25723**	2|203682||||	4.76934
**k__Bacteria|p__Actinobacteria|c__Actinomycetia|o__Candidatus_Nanopelagicales|f__Candidatus_Nanopelagicaceae|g__Candidatus_Nanopelagicus**	2|201174|1760|2039638|2162846|2039639	1.42626

**k__Bacteria|p__Actinobacteria|c__Actinomycetia|o__Candidatus_Nanopelagicales|f__Candidatus_Nanopelagicaceae|g__Candidatus_Planktophila**	2|201174|1760|2039638|2162846|622681	1.1383
**k__Bacteria|p__Bacteroidetes|c__CFGB40560|o__OFGB40560|f__FGB40560|g__GGB43022**	2|976||||	1.08541
**k__Bacteria|p__Bacteroidetes|c__CFGB18754|o__OFGB18754|f__FGB18754|g__GGB46527**	2|976||||	1.01439
**k__Bacteria|p__Actinobacteria|c__Actinomycetia|o__Actinomycetia_unclassified|f__Actinomycetia_unclassified|g__Actinomycetia_unclassified**	2|201174|1760|||	0.99582
**k__Bacteria|p__Proteobacteria|c__Betaproteobacteria|o__Nitrosomonadales|f__Nitrosomonadaceae|g__Nitrosospira**	2|1224|28216|32003|206379|35798	0.82594
**k__Bacteria|p__Actinobacteria|c__CFGB46334|o__OFGB46334|f__FGB46334|g__GGB46334**	2|201174||||	0.6312
**k__Bacteria|p__Proteobacteria|c__Betaproteobacteria|o__Burkholderiales|f__Burkholderiaceae|g__GGB26028**	2|1224|28216|80840|119060|	0.44443
**k__Bacteria|p__Proteobacteria|c__Betaproteobacteria|o__Betaproteobacteria_unclassified|f__Betaproteobacteria_unclassified|g__Betaproteobacteria_unclassified**	2|1224|28216|||	0.18702
**k__Bacteria|p__Proteobacteria|c__CFGB42731|o__OFGB42731|f__FGB42731|g__GGB47400**	2|1224||||	0.12265
**k__Bacteria|p__Proteobacteria|c__Betaproteobacteria|o__Nitrosomonadales|f__Methylophilaceae|g__Methylotenera**	2|1224|28216|32003|32011|359407	0.10793
**k__Bacteria|p__Actinobacteria|c__CFGB13659|o__OFGB13659|f__FGB13659|g__GGB23686**	2|201174||||	0.07377
**k__Bacteria|p__Actinobacteria|c__CFGB1083|o__OFGB1083|f__FGB1083|g__GGB24856**	2|201174||||	0.05903
**k__Bacteria|p__Actinobacteria|c__CFGB34754|o__OFGB34754|f__FGB34754|g__GGB34754**	2|201174||||	0.03313
**k__Bacteria|p__Bacteroidetes|c__CFGB41865|o__OFGB41865|f__FGB41865|g__GGB70913**	2|976||||	0.02749
**k__Bacteria|p__Bacteroidetes|c__Sphingobacteriia|o__Sphingobacteriales|f__Sphingobacteriaceae|g__Daejeonella**	2|976|117747|200666|84566|2762385	0.02278
**k__Bacteria|p__Proteobacteria|c__Betaproteobacteria|o__Nitrosomonadales|f__Nitrosomonadaceae|g__Nitrosomonas**	2|1224|28216|32003|206379|914	0.02121
**k__Bacteria|p__Nitrospirae|c__Nitrospira|o__Nitrospirales|f__Nitrospiraceae|g__Nitrospira**	2|40117|203693|189778|189779|1234	0.01845
**k__Bacteria|p__Actinobacteria|c__Actinomycetia|o__Bifidobacteriales|f__Bifidobacteriaceae|g__Gardnerella**	2|201174|1760|85004|31953|2701	0.00903
**k__Bacteria|p__Proteobacteria|c__Gammaproteobacteria|o__Pseudomonadales|f__Pseudomonadaceae|g__Pseudomonas|s__Pseudomonas_sp_PDM05**	2|1224|1236|72274|135621|286|2769301	45.63294
**k__Bacteria|p__Proteobacteria|c__Alphaproteobacteria|o__Alphaproteobacteria_unclassified|f__Alphaproteobacteria_unclassified|g__Alphaproteobacteria_unclassified|s__alpha_proteobacterium_SCGC_AAA028_D10**	2|1224|28211||||938641	13.08046
**k__Bacteria|p__Proteobacteria|c__Betaproteobacteria|o__Nitrosomonadales|f__Methylophilaceae|g__Candidatus_Methylopumilus|s__Candidatus_Methylopumilus_universalis**	2|1224|28216|32003|32011|1679002|2588536	12.84507
**k__Bacteria|p__Proteobacteria|c__Alphaproteobacteria|o__Pelagibacterales|f__Pelagibacterales_unclassified|g__Candidatus_Fonsibacter|s__Candidatus_Fonsibacter_ubiquis**	2|1224|28211|54526||2045213|1925548	9.60208
**k__Bacteria|p__Candidatus_Kryptonia|c__CFGB41117|o__OFGB41117|f__FGB41117|g__GGB75394|s__GGB75394_SGB103389**	2|1855361|||||	5.04592

**k__Bacteria|p__Planctomycetes|c__CFGB8429|o__OFGB8429|f__FGB8429|g__GGB25723|s__GGB25723_SGB84803**	2|203682||||	4.76934
**k__Bacteria|p__Actinobacteria|c__Actinomycetia|o__Candidatus_Nanopelagicales|f__Candidatus_Nanopelagicaceae|g__Candidatus_Nanopelagicus|s__Candidatus_Nanopelagicus_limnes**	2|201174|1760|2039638|2162846|2039639|1884634	1.22748
**k__Bacteria|p__Bacteroidetes|c__CFGB40560|o__OFGB40560|f__FGB40560|g__GGB43022|s__GGB43022_SGB60257**	2|976||||	1.08541
**k__Bacteria|p__Bacteroidetes|c__CFGB18754|o__OFGB18754|f__FGB18754|g__GGB46527|s__GGB46527_SGB64388**	2|976||||	1.01439
**k__Bacteria|p__Proteobacteria|c__Betaproteobacteria|o__Nitrosomonadales|f__Nitrosomonadaceae|g__Nitrosospira|s__Nitrosospira_sp_Nsp13**	2|1224|28216|32003|206379|35798|1855332	0.82594
**k__Bacteria|p__Actinobacteria|c__Actinomycetia|o__Actinomycetia_unclassified|f__Actinomycetia_unclassified|g__Actinomycetia_unclassified|s__Actinomycetia_unclassified_SGB64102**	2|201174|1760||||	0.70635
**k__Bacteria|p__Actinobacteria|c__CFGB46334|o__OFGB46334|f__FGB46334|g__GGB46334|s__GGB46334_SGB35380**	2|201174||||	0.6312
**k__Bacteria|p__Actinobacteria|c__Actinomycetia|o__Candidatus_Nanopelagicales|f__Candidatus_Nanopelagicaceae|g__Candidatus_Planktophila|s__Candidatus_Planktophila_vernalis**	2|201174|1760|2039638|2162846|622681|1884907	0.62978
**k__Bacteria|p__Proteobacteria|c__Gammaproteobacteria|o__Pseudomonadales|f__Pseudomonadaceae|g__Pseudomonas|s__Pseudomonas_laurylsulfatiphila**	2|1224|1236|72274|135621|286|2011015	0.5327
**k__Bacteria|p__Actinobacteria|c__Actinomycetia|o__Candidatus_Nanopelagicales|f__Candidatus_Nanopelagicaceae|g__Candidatus_Planktophila|s__Candidatus_Planktophila_lacus**	2|201174|1760|2039638|2162846|622681|1884913	0.50853
**k__Bacteria|p__Proteobacteria|c__Betaproteobacteria|o__Burkholderiales|f__Burkholderiaceae|g__GGB26028|s__GGB26028_SGB38032**	2|1224|28216|80840|119060||	0.44443
**k__Bacteria|p__Actinobacteria|c__Actinomycetia|o__Actinomycetia_unclassified|f__Actinomycetia_unclassified|g__Actinomycetia_unclassified|s__actinobacterium_SCGC_AAA028_A23**	2|201174|1760||||932036	0.28947
**k__Bacteria|p__Proteobacteria|c__Betaproteobacteria|o__Nitrosomonadales|f__Methylophilaceae|g__Candidatus_Methylopumilus|s__Candidatus_Methylopumilus_rimovensis**	2|1224|28216|32003|32011|1679002|2588535	0.24726
**k__Bacteria|p__Actinobacteria|c__Actinomycetia|o__Candidatus_Nanopelagicales|f__Candidatus_Nanopelagicaceae|g__Candidatus_Nanopelagicus|s__Candidatus_Nanopelagicus_abundans**	2|201174|1760|2039638|2162846|2039639|1884916	0.19877
**k__Bacteria|p__Proteobacteria|c__Betaproteobacteria|o__Betaproteobacteria_unclassified|f__Betaproteobacteria_unclassified|g__Betaproteobacteria_unclassified|s__beta_proteobacterium_SCGC_AAA027_K21**	2|1224|28216||||938785	0.18702
**k__Bacteria|p__Proteobacteria|c__CFGB42731|o__OFGB42731|f__FGB42731|g__GGB47400|s__GGB47400_SGB65360**	2|1224||||	0.12265
**k__Bacteria|p__Proteobacteria|c__Betaproteobacteria|o__Nitrosomonadales|f__Methylophilaceae|g__Methylotenera|s__Methylotenera_mobilis**	2|1224|28216|32003|32011|359407|359408	0.10793
**k__Bacteria|p__Actinobacteria|c__CFGB13659|o__OFGB13659|f__FGB13659|g__GGB23686|s__GGB23686_SGB82209**	2|201174||||	0.07377
**k__Bacteria|p__Actinobacteria|c__CFGB1083|o__OFGB1083|f__FGB1083|g__GGB24856|s__GGB24856_SGB81948**	2|201174||||	0.05903
**k__Bacteria|p__Actinobacteria|c__CFGB34754|o__OFGB34754|f__FGB34754|g__GGB34754|s__GGB34754_SGB82226**	2|201174||||	0.03313
**k__Bacteria|p__Bacteroidetes|c__CFGB41865|o__OFGB41865|f__FGB41865|g__GGB70913|s__GGB70913_SGB95372**	2|976||||	0.02749
**k__Bacteria|p__Bacteroidetes|c__Sphingobacteriia|o__Sphingobacteriales|f__Sphingobacteriaceae|g__Daejeonella|s__Daejeonella_rubra**	2|976|117747|200666|84566|2762385|990371	0.02278

<table>
<tr><td>k__Bacteria|p__Proteobacteria|c__Betaproteobacteria|o__Nitrosomonadales|f__Nitrosomonadaceae|g__Nitrosomonas|s__Nitrosomonas_sp_Nm84</td><td>2|1224|28216|32003|206379|914|200124</td><td>0.02121</td></tr>
<tr><td>k__Bacteria|p__Nitrospirae|c__Nitrospira|o__Nitrospirales|f__Nitrospiraceae|g__Nitrospira|s__Nitrospira_lenta</td><td>2|40117|203693|189778|189779|1234|1436998</td><td>0.01845</td></tr>
<tr><td>k__Bacteria|p__Actinobacteria|c__Actinomycetia|o__Bifidobacteriales|f__Bifidobacteriaceae|g__Gardnerella|s__Gardnerella_vaginalis</td><td>2|201174|1760|85004|31953|2701|2702</td><td>0.00903</td></tr>
<tr><td>k__Bacteria|p__Proteobacteria|c__Gammaproteobacteria|o__Pseudomonadales|f__Pseudomonadaceae|g__Pseudomonas|s__Pseudomonas_sp_PDM05|t__SGB89067</td><td>2|1224|1236|72274|135621|286|2769301|</td><td>45.63294</td></tr>
<tr><td>k__Bacteria|p__Proteobacteria|c__Alphaproteobacteria|o__Alphaproteobacteria_unclassified|f__Alphaproteobacteria_unclassified|g__Alphaproteobacteria_unclassified|s__alpha_proteobacterium_SCGC_AAA028_D10|t__SGB50895_group</td><td>2|1224|28211||||938641|</td><td>13.08046</td></tr>
<tr><td>k__Bacteria|p__Proteobacteria|c__Betaproteobacteria|o__Nitrosomonadales|f__Methylophilaceae|g__Candidatus_Methylopumilus|s__Candidatus_Methylopumilus_universalis|t__SGB38034</td><td>2|1224|28216|32003|32011|1679002|2588536|</td><td>12.84507</td></tr>
<tr><td>k__Bacteria|p__Proteobacteria|c__Alphaproteobacteria|o__Pelagibacterales|f__Pelagibacterales_unclassified|g__Candidatus_Fonsibacter|s__Candidatus_Fonsibacter_ubiquis|t__SGB28829</td><td>2|1224|28211|54526||2045213|1925548|</td><td>9.60208</td></tr>
<tr><td>k__Bacteria|p__Candidatus_Kryptonia|c__CFGB41117|o__OFGB41117|f__FGB41117|g__GGB75394|s__GGB75394_SGB103389|t__SGB103389</td><td>2|1855361|||||</td><td>5.04592</td></tr>
<tr><td>k__Bacteria|p__Planctomycetes|c__CFGB8429|o__OFGB8429|f__FGB8429|g__GGB25723|s__GGB25723_SGB84803|t__SGB84803</td><td>2|203682|||||</td><td>4.76934</td></tr>
<tr><td>k__Bacteria|p__Actinobacteria|c__Actinomycetia|o__Candidatus_Nanopelagicales|f__Candidatus_Nanopelagicaceae|g__Candidatus_Nanopelagicus|s__Candidatus_Nanopelagicus_limnes|t__SGB5711</td><td>2|201174|1760|2039638|2162846|2039639|1884634|</td><td>1.22748</td></tr>
<tr><td>k__Bacteria|p__Bacteroidetes|c__CFGB40560|o__OFGB40560|f__FGB40560|g__GGB43022|s__GGB43022_SGB60257|t__SGB60257</td><td>2|976|||||</td><td>1.08541</td></tr>
<tr><td>k__Bacteria|p__Bacteroidetes|c__CFGB18754|o__OFGB18754|f__FGB18754|g__GGB46527|s__GGB46527_SGB64388|t__SGB64388</td><td>2|976|||||</td><td>1.01439</td></tr>
<tr><td>k__Bacteria|p__Proteobacteria|c__Betaproteobacteria|o__Nitrosomonadales|f__Nitrosomonadaceae|g__Nitrosospira|s__Nitrosospira_sp_Nsp13|t__SGB13341</td><td>2|1224|28216|32003|206379|35798|1855332|</td><td>0.82594</td></tr>
<tr><td>k__Bacteria|p__Actinobacteria|c__Actinomycetia|o__Actinomycetia_unclassified|f__Actinomycetia_unclassified|g__Actinomycetia_unclassified|s__Actinomycetia_unclassified_SGB64102|t__SGB64102</td><td>2|201174|1760||||</td><td>0.70635</td></tr>
<tr><td>k__Bacteria|p__Actinobacteria|c__CFGB46334|o__OFGB46334|f__FGB46334|g__GGB46334|s__GGB46334_SGB35380|t__SGB35380</td><td>2|201174|||||</td><td>0.6312</td></tr>
<tr><td>k__Bacteria|p__Actinobacteria|c__Actinomycetia|o__Candidatus_Nanopelagicales|f__Candidatus_Nanopelagicaceae|g__Candidatus_Planktophila|s__Candidatus_Planktophila_vernalis|t__SGB5705</td><td>2|201174|1760|2039638|2162846|622681|1884907|</td><td>0.62978</td></tr>
<tr><td>k__Bacteria|p__Proteobacteria|c__Gammaproteobacteria|o__Pseudomonadales|f__Pseudomonadaceae|g__Pseudomonas|s__Pseudomonas_laurylsulfatiphila|t__SGB12221</td><td>2|1224|1236|72274|135621|286|2011015|</td><td>0.5327</td></tr>
<tr><td>k__Bacteria|p__Actinobacteria|c__Actinomycetia|o__Candidatus_Nanopelagicales|f__Candidatus_Nanopelagicaceae|g__Candidatus_Planktophila|s__Candidatus_Planktophila_lacus|t__SGB5706</td><td>2|201174|1760|2039638|2162846|622681|1884913|</td><td>0.50853</td></tr>
</table>

**k__Bacteria|p__Proteobacteria|c__Betaproteobacteria|o__Burkholderiales|f__Burkholderiaceae|g__GGB26028|s__GGB26028_SGB38032|t__SGB38032**	2|1224|28216|80840|119060|||	0.44443
**k__Bacteria|p__Actinobacteria|c__Actinomycetia|o__Actinomycetia_unclassified|f__Actinomycetia_unclassified|g__Actinomycetia_unclassified|s__actinobacterium_SCGC_AAA028_A23|t__SGB5713**	2|201174|1760||||932036|	0.28947
**k__Bacteria|p__Proteobacteria|c__Betaproteobacteria|o__Nitrosomonadales|f__Methylophilaceae|g__Candidatus_Methylopumilus|s__Candidatus_Methylopumilus_rimovensis|t__SGB80878**	2|1224|28216|32003|32011|1679002|2588535|	0.24726
**k__Bacteria|p__Actinobacteria|c__Actinomycetia|o__Candidatus_Nanopelagicales|f__Candidatus_Nanopelagicaceae|g__Candidatus_Nanopelagicus|s__Candidatus_Nanopelagicus_abundans|t__SGB5714**	2|201174|1760|2039638|2162846|2039639|1884916|	0.19877
**k__Bacteria|p__Proteobacteria|c__Betaproteobacteria|o__Betaproteobacteria_unclassified|f__Betaproteobacteria_unclassified|g__Betaproteobacteria_unclassified|s__beta_proteobacterium_SCGC_AAA027_K21|t__SGB57451**	2|1224|28216||||938785|	0.18702
**k__Bacteria|p__Proteobacteria|c__CFGB42731|o__OFGB42731|f__FGB42731|g__GGB47400|s__GGB47400_SGB65360|t__SGB65360**	2|1224||||||	0.12265
**k__Bacteria|p__Proteobacteria|c__Betaproteobacteria|o__Nitrosomonadales|f__Methylophilaceae|g__Methylotenera|s__Methylotenera_mobilis|t__SGB30611**	2|1224|28216|32003|32011|359407|359408|	0.10793
**k__Bacteria|p__Actinobacteria|c__CFGB13659|o__OFGB13659|f__FGB13659|g__GGB23686|s__GGB23686_SGB82209|t__SGB82209**	2|201174||||||	0.07377
**k__Bacteria|p__Actinobacteria|c__CFGB1083|o__OFGB1083|f__FGB1083|g__GGB24856|s__GGB24856_SGB81948|t__SGB81948**	2|201174||||||	0.05903
**k__Bacteria|p__Actinobacteria|c__CFGB34754|o__OFGB34754|f__FGB34754|g__GGB34754|s__GGB34754_SGB82226|t__SGB82226**	2|201174||||||	0.03313
**k__Bacteria|p__Bacteroidetes|c__CFGB41865|o__OFGB41865|f__FGB41865|g__GGB70913|s__GGB70913_SGB95372|t__SGB95372**	2|976||||||	0.02749
**k__Bacteria|p__Bacteroidetes|c__Sphingobacteriia|o__Sphingobacteriales|f__Sphingobacteriaceae|g__Daejeonella|s__Daejeonella_rubra|t__SGB3127**	2|976|117747|200666|84566|2762385|990371|	0.02278
**k__Bacteria|p__Proteobacteria|c__Betaproteobacteria|o__Nitrosomonadales|f__Nitrosomonadaceae|g__Nitrosomonas|s__Nitrosomonas_sp_Nm84|t__SGB24812**	2|1224|28216|32003|206379|914|200124|	0.02121
**k__Bacteria|p__Nitrospirae|c__Nitrospira|o__Nitrospirales|f__Nitrospiraceae|g__Nitrospira|s__Nitrospira_lenta|t__SGB22161**	2|40117|203693|189778|189779|1234|1436998|	0.01845
**k__Bacteria|p__Actinobacteria|c__Actinomycetia|o__Bifidobacteriales|f__Bifidobacteriaceae|g__Gardnerella|s__Gardnerella_vaginalis|t__SGB17307**	2|201174|1760|85004|31953|2701|2702|	0.00903

Table S6. Presence of Xenobiotic pathways in the Rustfontein Raw Summer samples, used to generate **Figure 5.**

# Pathway	output_RRS.assembled_Abundance
**PWY-5695: inosine 5'-phosphate degradation**	217,113828
**PWY-5695: inosine 5'-phosphate degradation|unclassified**	183,54416
**HISDEG-PWY: L-histidine degradation I**	137,458236
**HISDEG-PWY: L-histidine degradation I|unclassified**	125,058662
**PWY0-1296: purine ribonucleosides degradation|unclassified**	115,483681
**PWY-6353: purine nucleotides degradation II (aerobic)**	72,9491562
**PWY-6353: purine nucleotides degradation II (aerobic)|unclassified**	66,1476915
**PWY-6901: superpathway of glucose and xylose degradation**	62,0966261
**SALVADEHYPOX-PWY: adenosine nucleotides degradation II**	55,1892881
**PWY-6317: D-galactose degradation I (Leloir pathway)**	53,2813345
**SALVADEHYPOX-PWY: adenosine nucleotides degradation II|unclassified**	49,4637409
**PWY-6608: guanosine nucleotides degradation III**	47,042087
**PWY-6608: guanosine nucleotides degradation III|unclassified**	45,7905272
**PWY0-1297: superpathway of purine deoxyribonucleosides degradation**	44,789962
**PWY0-1297: superpathway of purine deoxyribonucleosides degradation|unclassified**	40,9495499
**PWY-6317: D-galactose degradation I (Leloir pathway)|unclassified**	39,1436785
**PWY0-1298: superpathway of pyrimidine deoxyribonucleosides degradation**	35,5334125
**PWY-6901: superpathway of glucose and xylose degradation|unclassified**	34,3089276
**PWY-8187: L-arginine degradation XIII (reductive Stickland reaction)**	34,3030292
**PWY0-1298: superpathway of pyrimidine deoxyribonucleosides degradation|unclassified**	32,4558035
**PWY-6606: guanosine nucleotides degradation II**	32,1009935
**PWY-6902: chitin degradation II (Vibrio)**	27,6300431
**PWY-7237: myo-, chiro- and scyllo-inositol degradation**	26,5970104
**PWY66-389: phytol degradation**	26,4402363
**PWY-5028: L-histidine degradation II**	25,87498
**PWY-7209: superpathway of pyrimidine ribonucleosides degradation**	23,1098616
**PWY-561: superpathway of glyoxylate cycle and fatty acid degradation**	22,0796149

<table>
<tr><td>PWY0-1296: purine ribonucleosides degradation|g__Candidatus_Nanopelagicus.s__Candidatus_Nanopelagicus_limnes</td><td>18,1610534</td></tr>
<tr><td>PWY-5030: L-histidine degradation III</td><td>17,739034</td></tr>
<tr><td>PWY-5030: L-histidine degradation III|unclassified</td><td>17,6255308</td></tr>
<tr><td>PWY-8187: L-arginine degradation XIII (reductive Stickland reaction)|unclassified</td><td>16,7955539</td></tr>
<tr><td>PWY-5941: glycogen degradation II</td><td>16,1507005</td></tr>
<tr><td>PWY-6606: guanosine nucleotides degradation II|unclassified</td><td>15,7457716</td></tr>
<tr><td>AST-PWY: L-arginine degradation II (AST pathway)</td><td>15,1840516</td></tr>
<tr><td>PWY-5130: 2-oxobutanoate degradation I</td><td>14,7162493</td></tr>
<tr><td>PWY-5028: L-histidine degradation II|unclassified</td><td>13,5438764</td></tr>
<tr><td>PWY-561: superpathway of glyoxylate cycle and fatty acid degradation|unclassified</td><td>13,4950172</td></tr>
<tr><td>12DICHLORETHDEG-PWY: 1,2-dichloroethane degradation</td><td>13,3530626</td></tr>
<tr><td>PWY66-389: phytol degradation|unclassified</td><td>10,664841</td></tr>
<tr><td>PWY-6328: L-lysine degradation X</td><td>9,83357453</td></tr>
<tr><td>PROTOCATECHUATE-ORTHO-CLEAVAGE-PWY: protocatechuate degradation II (ortho-cleavage pathway)</td><td>8,77163829</td></tr>
<tr><td>PWY-7209: superpathway of pyrimidine ribonucleosides degradation|unclassified</td><td>7,83496379</td></tr>
<tr><td>PWY-5941: glycogen degradation II|unclassified</td><td>7,43495607</td></tr>
<tr><td>12DICHLORETHDEG-PWY: 1,2-dichloroethane degradation|unclassified</td><td>7,21308187</td></tr>
<tr><td>CATECHOL-ORTHO-CLEAVAGE-PWY: catechol degradation to β-ketoadipate</td><td>7,02087724</td></tr>
<tr><td>PWY-5417: catechol degradation III (ortho-cleavage pathway)</td><td>6,98731821</td></tr>
<tr><td>PWY-5431: aromatic compounds degradation via β-ketoadipate</td><td>6,98731821</td></tr>
<tr><td>PWY-6902: chitin degradation II (Vibrio)|unclassified</td><td>6,71395507</td></tr>
<tr><td>P161-PWY: acetylene degradation (anaerobic)</td><td>6,65527187</td></tr>
<tr><td>PWY-5130: 2-oxobutanoate degradation I|unclassified</td><td>6,49951821</td></tr>
<tr><td>P161-PWY: acetylene degradation (anaerobic)|unclassified</td><td>6,41120729</td></tr>
<tr><td>PWY-8187: L-arginine degradation XIII (reductive Stickland reaction)|g__Pseudomonas.s__Pseudomonas_fluorescens_group</td><td>6,32703555</td></tr>
<tr><td>PWY-7237: myo-, chiro- and scyllo-inositol degradation|unclassified</td><td>6,23931886</td></tr>
<tr><td>PWY-6507: 4-deoxy-L-threo-hex-4-enopyranuronate degradation</td><td>5,49530605</td></tr>
<tr><td>PWY-6507: 4-deoxy-L-threo-hex-4-enopyranuronate degradation|unclassified</td><td>5,48435396</td></tr>
<tr><td>ALLANTOINDEG-PWY: superpathway of allantoin degradation in yeast</td><td>5,13128516</td></tr>
<tr><td>PWY66-389: phytol degradation|g__Pseudomonas.s__Pseudomonas_fluorescens_group</td><td>5,08356429</td></tr>
</table>

**PWY0-1296: purine ribonucleosides degradation|g__Candidatus_Nanopelagicus.s__Candidatus_Nanopelagicus_abundans**	4,69252319
**CATECHOL-ORTHO-CLEAVAGE-PWY: catechol degradation to β-ketoadipate|g__Pseudomonas.s__Pseudomonas_fluorescens_group**	3,70839926
**TYRFUMCAT-PWY: L-tyrosine degradation I**	3,34339774
**PROTOCATECHUATE-ORTHO-CLEAVAGE-PWY: protocatechuate degradation II (ortho-cleavage pathway)|g__Pseudomonas.s__Pseudomonas_simiae**	3,00052482
**TYRFUMCAT-PWY: L-tyrosine degradation I|unclassified**	2,28949483
**PROTOCATECHUATE-ORTHO-CLEAVAGE-PWY: protocatechuate degradation II (ortho-cleavage pathway)|g__Pseudomonas.s__Pseudomonas_fluorescens_group**	2,08006468
**PROTOCATECHUATE-ORTHO-CLEAVAGE-PWY: protocatechuate degradation II (ortho-cleavage pathway)|unclassified**	1,92110828

Table S7. Presence of Xenobiotic Pathways in the Rustfontein Raw Winter samples, used to generate Figure 5.

# Pathway	output_RRW2.assembled_Abundance
**HISDEG-PWY: L-histidine degradation I**	276.2425603392
**HISDEG-PWY: L-histidine degradation I|unclassified**	130.2993962004
**HISDEG-PWY: L-histidine degradation I|g__Acinetobacter.s__Acinetobacter_bohemicus**	127.9206582646
**HISDEG-PWY: L-histidine degradation I|g__Pseudomonas.s__Pseudomonas_fluorescens_group**	11.5128638428
**HISDEG-PWY: L-histidine degradation I|g__Bacillus.s__Bacillus_cereus_group**	5.5284707526
**PWY-561: superpathway of glyoxylate cycle and fatty acid degradation**	221.2151210343
**PWY-561: superpathway of glyoxylate cycle and fatty acid degradation|unclassified**	30.0438497613
**PWY0-1296: purine ribonucleosides degradation**	205.6525913961
**PWY0-1296: purine ribonucleosides degradation|unclassified**	106.4137798310
**PWY0-1296: purine ribonucleosides degradation|g__Candidatus_Nanopelagicus.s__Candidatus_Nanopelagicus_limnes**	27.5609839007
**PWY0-1296: purine ribonucleosides degradation|g__Candidatus_Nanopelagicus.s__Candidatus_Nanopelagicus_abundans**	5.8558907989
**AST-PWY: L-arginine degradation II (AST pathway)**	164.7198403204
**AST-PWY: L-arginine degradation II (AST pathway)|g__Acinetobacter.s__Acinetobacter_bohemicus**	128.9470318211
**AST-PWY: L-arginine degradation II (AST pathway)|unclassified**	6.1613971328

**PWY0-1297: superpathway of purine deoxyribonucleosides degradation**	134.4609961855
**PWY0-1297: superpathway of purine deoxyribonucleosides degradation|unclassified**	34.8122472198
**PWY-6901: superpathway of glucose and xylose degradation**	118.7932199777
**PWY-6901: superpathway of glucose and xylose degradation|unclassified**	70.1517964970
**PWY-6901: superpathway of glucose and xylose degradation|g__Pseudomonas.s__Pseudomonas_fluorescens_group**	14.6693024379
**PWY0-1298: superpathway of pyrimidine deoxyribonucleosides degradation**	117.7260993563
**PWY0-1298: superpathway of pyrimidine deoxyribonucleosides degradation|unclassified**	18.3528806403
**PWY-6353: purine nucleotides degradation II (aerobic)**	101.1044863192
**PWY-6353: purine nucleotides degradation II (aerobic)|unclassified**	42.4412499548
**METHGLYUT-PWY: superpathway of methylglyoxal degradation**	87.7372129814
**GLUCOSE1PMETAB-PWY: glucose and glucose-1-phosphate degradation**	82.9910362962
**GLUCOSE1PMETAB-PWY: glucose and glucose-1-phosphate degradation|unclassified**	61.1781786198
**GLUCOSE1PMETAB-PWY: glucose and glucose-1-phosphate degradation|g__Pseudomonas.s__Pseudomonas_fluorescens_group**	16.5402204706
**GLUCOSE1PMETAB-PWY: glucose and glucose-1-phosphate degradation|g__Pseudomonas.s__Pseudomonas_putida_group**	1.4175478652
**PWY-6317: D-galactose degradation I (Leloir pathway)**	82.4192981228
**PWY-6317: D-galactose degradation I (Leloir pathway)|unclassified**	42.4526952295
**PWY66-389: phytol degradation**	81.1960602072
**PWY66-389: phytol degradation|unclassified**	49.9697520932
**PWY66-389: phytol degradation|g__Pseudomonas.s__Pseudomonas_fluorescens_group**	4.7791506800
**PPGPPMET-PWY: ppGpp metabolism**	79.5152556661
**PPGPPMET-PWY: ppGpp metabolism|unclassified**	65.2340682727
**SALVADEHYPOX-PWY: adenosine nucleotides degradation II**	74.9438683502
**SALVADEHYPOX-PWY: adenosine nucleotides degradation II|unclassified**	30.5592859513
**PWY-6608: guanosine nucleotides degradation III**	67.1834740013
**PWY-6608: guanosine nucleotides degradation III|unclassified**	27.0950853740
**PWY-8187: L-arginine degradation XIII (reductive Stickland reaction)**	63.8243572805
**PWY-8187: L-arginine degradation XIII (reductive Stickland reaction)|unclassified**	22.2715762852

**PWY-8187: L-arginine degradation XIII (reductive Stickland reaction)|g__Pseudomonas.s__Pseudomonas_fluorescens_group**	7.1411070131
**P161-PWY: acetylene degradation (anaerobic)**	61.6430467758
**P161-PWY: acetylene degradation (anaerobic)|unclassified**	10.7374541510
**PWY-6902: chitin degradation II (Vibrio)**	53.3646237709
**PWY-6902: chitin degradation II (Vibrio)|unclassified**	27.0006463173
**PWY-6606: guanosine nucleotides degradation II**	48.6684968636
**PWY-6606: guanosine nucleotides degradation II|unclassified**	9.5113405962
**PWY-6607: guanosine nucleotides degradation I**	48.6684968636
**PWY-6607: guanosine nucleotides degradation I|unclassified**	4.2467551885
**PWY-5941: glycogen degradation II**	38.5788434995
**PWY-5941: glycogen degradation II|unclassified**	27.6873546272
**PWY-5941: glycogen degradation II|g__Pseudomonas.s__Pseudomonas_fluorescens_group**	8.6344336365
**PWY-5941: glycogen degradation II|g__Pseudomonas.s__Pseudomonas_putida**	0.5782911046
**PWY-6595: superpathway of guanosine nucleotides degradation (plants)**	31.9639348245
**PWY-6595: superpathway of guanosine nucleotides degradation (plants)|unclassified**	3.8737677095
**CATECHOL-ORTHO-CLEAVAGE-PWY: catechol degradation to β-ketoadipate**	30.6959605427
**CATECHOL-ORTHO-CLEAVAGE-PWY: catechol degradation to β-ketoadipate|unclassified**	13.0801751014
**PROTOCATECHUATE-ORTHO-CLEAVAGE-PWY: protocatechuate degradation II (ortho-cleavage pathway)**	29.0810572810
**PROTOCATECHUATE-ORTHO-CLEAVAGE-PWY: protocatechuate degradation II (ortho-cleavage pathway)|unclassified**	11.3106462011
**PWY-5028: L-histidine degradation II**	27.9291998288
**PWY-5028: L-histidine degradation II|unclassified**	20.4218212106
**PWY-5028: L-histidine degradation II|g__Pseudomonas.s__Pseudomonas_fluorescens_group**	5.3759881559
**PWY-7242: D-fructuronate degradation**	27.6451836808
**PWY-7242: D-fructuronate degradation|unclassified**	12.4893598921
**PWY-6328: L-lysine degradation X**	27.4860566228
**PWY-6328: L-lysine degradation X|g__Pseudomonas.s__Pseudomonas_fluorescens_group**	9.3281154171
**PWY-5180: toluene degradation I (aerobic) (via o-cresol)**	25.5778330510

**PWY-5180: toluene degradation I (aerobic) (via o-cresol)|unclassified**	6.9292308072
**PWY-6507: 4-deoxy-L-threo-hex-4-enopyranuronate degradation**	24.9944730755
**PWY-6507: 4-deoxy-L-threo-hex-4-enopyranuronate degradation|unclassified**	11.9183355287
**PWY-7237: myo-, chiro- and scyllo-inositol degradation**	22.9294622216
**PWY-7237: myo-, chiro- and scyllo-inositol degradation|unclassified**	18.9665128261
**LEU-DEG2-PWY: L-leucine degradation I**	21.6857854520
**PWY-7209: superpathway of pyrimidine ribonucleosides degradation**	19.9870743693
**PWY-7209: superpathway of pyrimidine ribonucleosides degradation|unclassified**	7.5961657902
**PWY-5417: catechol degradation III (ortho-cleavage pathway)**	19.7389154641
**PWY-5417: catechol degradation III (ortho-cleavage pathway)|unclassified**	8.1955291516
**PWY-5431: aromatic compounds degradation via β-ketoadipate**	19.7389154641
**PWY-5431: aromatic compounds degradation via β-ketoadipate|unclassified**	8.1955291516
**DENITRIFICATION-PWY: nitrate reduction I (denitrification)**	16.7578936976
**DENITRIFICATION-PWY: nitrate reduction I (denitrification)|unclassified**	16.2806810845
**TYRFUMCAT-PWY: L-tyrosine degradation I**	15.5982154550
**TYRFUMCAT-PWY: L-tyrosine degradation I|unclassified**	13.9655748226
**PWY-6182: superpathway of salicylate degradation**	12.6462989025
**PWY-6182: superpathway of salicylate degradation|unclassified**	4.3123524425
**PWY-6731: starch degradation III**	12.0608718601
**PWY-6731: starch degradation III|unclassified**	11.5103268443
**12DICHLORETHDEG-PWY: 1,2-dichloroethane degradation**	11.9432459416
**12DICHLORETHDEG-PWY: 1,2-dichloroethane degradation|unclassified**	10.2264183397
**PWY-6185: 4-methylcatechol degradation (ortho cleavage)**	11.3876996624
**PWY-6185: 4-methylcatechol degradation (ortho cleavage)|unclassified**	3.3306885894
**PWY-5415: catechol degradation I (meta-cleavage pathway)**	9.0571285348
**PWY-5415: catechol degradation I (meta-cleavage pathway)|unclassified**	2.7081540820
**ORNDEG-PWY: superpathway of ornithine degradation**	6.5204240130
**GALACTUROCAT-PWY: D-galacturonate degradation I**	6.2305033023
**GALACTUROCAT-PWY: D-galacturonate degradation I|unclassified**	5.6109636614
**PWY-8131: 5'-deoxyadenosine degradation II**	5.6175390545

**PWY-6309: L-tryptophan degradation XI (mammalian, via kynurenine)**	4.8051872387
**PWY-6309: L-tryptophan degradation XI (mammalian, via kynurenine)|unclassified**	4.8051872387

Table S8. Presence of Xenobiotic Pathways in the Welbedacht Raw Summer samples, used to generate Figure 5.

# **Pathway**	**output_WRS.assembled_Abundance**
**PWY-5695: inosine 5'-phosphate degradation**	242.6949971933
**PWY-5695: inosine 5'-phosphate degradation|unclassified**	188.5452290405
**PWY0-1296: purine ribonucleosides degradation**	240.2747754235
**PWY0-1296: purine ribonucleosides degradation|unclassified**	196.7700885286
**PWY0-1296: purine ribonucleosides degradation|g__Candidatus_Nanopelagicus.s__Candidatus_Nanopelagicus_limnes**	40.2174211442
**PWY0-1296: purine ribonucleosides degradation|g__Candidatus_Nanopelagicus.s__Candidatus_Nanopelagicus_abundans**	36.4009965762
**THRESYN-PWY: superpathway of L-threonine biosynthesis**	31.0772973613
**HISDEG-PWY: L-histidine degradation I**	111.2843984071
**HISDEG-PWY: L-histidine degradation I|unclassified**	116.2553846504
**PWY-6901: superpathway of glucose and xylose degradation**	102.2499250948
**PWY-6901: superpathway of glucose and xylose degradation|unclassified**	109.6510708930
**PWY-7237: myo-, chiro- and scyllo-inositol degradation|unclassified**	83.3425156530
**PWY-6902: chitin degradation II (Vibrio)**	83.3425156530
**PWY-6902: chitin degradation II (Vibrio)|unclassified**	81.8462627646
**PWY66-389: phytol degradation**	68.3037966013
**PWY66-389: phytol degradation|unclassified**	67.8539755691
**PWY-8187: L-arginine degradation XIII (reductive Stickland reaction)**	51.0784711669
**PWY-8187: L-arginine degradation XIII (reductive Stickland reaction)|unclassified**	48.9272864688
**PWY-6549: L-glutamine biosynthesis III**	48.2705854273
**PWY-6549: L-glutamine biosynthesis III|unclassified**	48.4153150345
**PWY-6317: D-galactose degradation I (Leloir pathway)**	45.2665889944
**PWY-6317: D-galactose degradation I (Leloir pathway)|unclassified**	48.1891025394
**PWY-7345: superpathway of anaerobic sucrose degradation**	43.0425541177

**PWY-7242: D-fructuronate degradation**	32.6782046976
**PWY-7242: D-fructuronate degradation|unclassified**	38.6434980484
**PWY-6507: 4-deoxy-L-threo-hex-4-enopyranuronate degradation**	38.6434980484
**PWY-6507: 4-deoxy-L-threo-hex-4-enopyranuronate degradation|unclassified**	37.9556570192
**PWY-5941: glycogen degradation II**	37.0266523285
**PWY-5941: glycogen degradation II|unclassified**	37.1036499281
**PWY-5651: L-tryptophan degradation to 2-amino-3-carboxymuconate semialdehyde**	36.1017083284
**PWY-5651: L-tryptophan degradation to 2-amino-3-carboxymuconate semialdehyde|unclassified**	36.8445868095
**AST-PWY: L-arginine degradation II (AST pathway)**	36.8445868095
**AST-PWY: L-arginine degradation II (AST pathway)|unclassified**	36.4202461884
**PWY-5130: 2-oxobutanoate degradation I**	36.4202461884
**PWY-5130: 2-oxobutanoate degradation I|unclassified**	35.4462863766
**PROTOCATECHUATE-ORTHO-CLEAVAGE-PWY: protocatechuate degradation II (ortho-cleavage pathway)**	35.0327298326
**PROTOCATECHUATE-ORTHO-CLEAVAGE-PWY: protocatechuate degradation II (ortho-cleavage pathway)|unclassified**	34.2894026450
**PWY0-1297: superpathway of purine deoxyribonucleosides degradation**	30.3437182084
**PWY0-1297: superpathway of purine deoxyribonucleosides degradation|unclassified**	30.3412012665
**PWY-5028: L-histidine degradation II**	29.9256924749
**PWY-5028: L-histidine degradation II|unclassified**	29.5399080386
**PWY-6328: L-lysine degradation X**	28.7160795623
**PWY-6328: L-lysine degradation X|unclassified**	26.9982933205
**TYRFUMCAT-PWY: L-tyrosine degradation I**	25.5689516278
**TYRFUMCAT-PWY: L-tyrosine degradation I|unclassified**	25.7243597487
**PWY-6353: purine nucleotides degradation II (aerobic)**	25.0364791374
**PWY-6353: purine nucleotides degradation II (aerobic)|unclassified**	25.4451274068
**PWY-7209: superpathway of pyrimidine ribonucleosides degradation**	23.3840483436
**PWY-7209: superpathway of pyrimidine ribonucleosides degradation|unclassified**	22.0556705407
**PWY0-1298: superpathway of pyrimidine deoxyribonucleosides degradation**	20.3022773020
**PWY0-1298: superpathway of pyrimidine deoxyribonucleosides degradation|unclassified**	19.9425355210
**P161-PWY: acetylene degradation (anaerobic)**	19.8831956100
**P161-PWY: acetylene degradation (anaerobic)|unclassified**	19.2968893482

**GALACTUROCAT-PWY: D-galacturonate degradation I**	19.2968893482
**GALACTUROCAT-PWY: D-galacturonate degradation I|unclassified**	17.7652823954
**LEU-DEG2-PWY: L-leucine degradation I**	17.6226773243
**LEU-DEG2-PWY: L-leucine degradation I|unclassified**	17.6295300850
**PWY-6608: guanosine nucleotides degradation III**	16.7598484598
**PWY-6608: guanosine nucleotides degradation III|unclassified**	17.5710439823
**PWY-5030: L-histidine degradation III**	17.1481985427
**PWY-5030: L-histidine degradation III|unclassified**	17.1309460459
**SALVADEHYPOX-PWY: adenosine nucleotides degradation II**	15.4029135857
**SALVADEHYPOX-PWY: adenosine nucleotides degradation II|unclassified**	15.2605935548
**ORNDEG-PWY: superpathway of ornithine degradation**	14.0801211210
**ORNDEG-PWY: superpathway of ornithine degradation|unclassified**	14.1093149404
**PWY-5384: sucrose degradation IV (sucrose phosphorylase)**	12.6047038115
**PWY-621: sucrose degradation III (sucrose invertase)**	10.1270332969
**PWY-5675: nitrate reduction V (assimilatory)**	10.1052930977
**PWY-5675: nitrate reduction V (assimilatory)|unclassified**	9.8606080019
**PWY-6606: guanosine nucleotides degradation II**	9.7731476559
**PWY-6606: guanosine nucleotides degradation II|unclassified**	9.4784095005
**PWY-6185: 4-methylcatechol degradation (ortho cleavage)**	9.1820305744
**PWY-6185: 4-methylcatechol degradation (ortho cleavage)|unclassified**	9.0594341533
**METHGLYUT-PWY: superpathway of methylglyoxal degradation**	7.5083118773
**METHGLYUT-PWY: superpathway of methylglyoxal degradation|unclassified**	6.9848424387

Table S9. Presence of Xenobiotic Pathways in the Welbedacht Raw Winter samples used to generate Figure 5.

# **Pathway**	**output_WRW.assembled_Abundance**
**PWY-5695: inosine 5'-phosphate degradation**	694.8764481003
**PWY-5695: inosine 5'-phosphate degradation|unclassified**	599.6263784470
**PWY-5695: inosine 5'-phosphate degradation|g__Pedobacter.s__Pedobacter_ruber**	5.9039429390
**PWY0-1296: purine ribonucleosides degradation**	589.9095360219
**PWY0-1296: purine ribonucleosides degradation|unclassified**	120.9063060362
**PWY0-1296: purine ribonucleosides degradation|g__Candidatus_Nanopelagicus.s__Candidatus_Nanopelagicus_limnes**	79.7346677439
**PWY0-1296: purine ribonucleosides degradation|g__Candidatus_Nanopelagicus.s__Candidatus_Nanopelagicus_abundans**	61.9981746350
**PWY0-1296: purine ribonucleosides degradation|g__Candidatus_Planktophila.s__Candidatus_Planktophila_lacus**	8.9917313214
**HISDEG-PWY: L-histidine degradation I**	486.3027535323
**HISDEG-PWY: L-histidine degradation I|unclassified**	480.2431124331
**PWY-6901: superpathway of glucose and xylose degradation**	465.1521757651
**PWY-6901: superpathway of glucose and xylose degradation|unclassified**	448.1465758222
**PWY-7237: myo-, chiro- and scyllo-inositol degradation**	360.2022900469
**PWY-7237: myo-, chiro- and scyllo-inositol degradation|unclassified**	360.2022900469
**PWY-8187: L-arginine degradation XIII (reductive Stickland reaction)**	339.3154416791
**PWY-8187: L-arginine degradation XIII (reductive Stickland reaction)|unclassified**	323.5701852587
**PWY-6902: chitin degradation II (Vibrio)**	298.4160340381
**PWY-6902: chitin degradation II (Vibrio)|unclassified**	293.9665816173
**PWY66-389: phytol degradation**	281.5536259777
**PWY66-389: phytol degradation|unclassified**	281.5536259777
**GLUCOSE1PMETAB-PWY: glucose and glucose-1-phosphate degradation**	206.8302094752
**GLUCOSE1PMETAB-PWY: glucose and glucose-1-phosphate degradation|unclassified**	202.7363549341
**PWY-5941: glycogen degradation II**	204.2062074919
**PWY-5941: glycogen degradation II|unclassified**	203.5299178301
**PWY-6353: purine nucleotides degradation II (aerobic)**	189.1238042368
**PWY-6353: purine nucleotides degradation II (aerobic)|unclassified**	147.8077782658
**PROTOCATECHUATE-ORTHO-CLEAVAGE-PWY: protocatechuate degradation II (ortho-cleavage pathway)**	186.6661472026

**PROTOCATECHUATE-ORTHO-CLEAVAGE-PWY: protocatechuate degradation II (ortho-cleavage pathway)|unclassified**	186.6661472026
**AST-PWY: L-arginine degradation II (AST pathway)**	173.3427277311
**AST-PWY: L-arginine degradation II (AST pathway)|unclassified**	173.3427277311
**PWY-5028: L-histidine degradation II**	156.3322464902
**PWY-5028: L-histidine degradation II|unclassified**	156.0427733073
**PWY-6317: D-galactose degradation I (Leloir pathway)**	150.2977428629
**PWY-6317: D-galactose degradation I (Leloir pathway)|unclassified**	115.5222361947
**PWY-101: photosynthesis light reactions**	145.1903552358
**SALVADEHYPOX-PWY: adenosine nucleotides degradation II**	140.0796320031
**SALVADEHYPOX-PWY: adenosine nucleotides degradation II|unclassified**	101.5024091050
**PWY-7345: superpathway of anaerobic sucrose degradation**	131.0365818102
**PWY-7345: superpathway of anaerobic sucrose degradation|unclassified**	128.2709901166
**PWY0-1297: superpathway of purine deoxyribonucleosides degradation**	121.9797186216
**PWY0-1297: superpathway of purine deoxyribonucleosides degradation|unclassified**	86.0704260215
**PWY-6608: guanosine nucleotides degradation III**	119.3022818633
**PWY-6608: guanosine nucleotides degradation III|unclassified**	117.6219157576
**PWY-7209: superpathway of pyrimidine ribonucleosides degradation**	116.1022009610
**PWY-7209: superpathway of pyrimidine ribonucleosides degradation|unclassified**	111.1096646592
**PWY-6328: L-lysine degradation X**	116.0889034708
**PWY-6328: L-lysine degradation X|unclassified**	116.0889034708
**PWY-7242: D-fructuronate degradation**	109.3066475614
**PWY-7242: D-fructuronate degradation|unclassified**	109.3066475614
**TYRFUMCAT-PWY: L-tyrosine degradation I**	93.0397243668
**TYRFUMCAT-PWY: L-tyrosine degradation I|unclassified**	93.0397243668
**PWY-6507: 4-deoxy-L-threo-hex-4-enopyranuronate degradation**	90.8560072543
**PWY-6507: 4-deoxy-L-threo-hex-4-enopyranuronate degradation|unclassified**	90.8560072543
**PWY0-1298: superpathway of pyrimidine deoxyribonucleosides degradation**	90.5231957990
**PWY0-1298: superpathway of pyrimidine deoxyribonucleosides degradation|unclassified**	87.7242595496
**PWY-6606: guanosine nucleotides degradation II**	81.6963856995
**PWY-6606: guanosine nucleotides degradation II|unclassified**	81.6400837802

**PWY-5651: L-tryptophan degradation to 2-amino-3-carboxymuconate semialdehyde**	64.2300954431
**PWY-5651: L-tryptophan degradation to 2-amino-3-carboxymuconate semialdehyde|unclassified**	63.7470952764
**LEU-DEG2-PWY: L-leucine degradation I**	49.4057113505
**LEU-DEG2-PWY: L-leucine degradation I|unclassified**	49.3844765060
**ORNDEG-PWY: superpathway of ornithine degradation**	47.5277366386
**ORNDEG-PWY: superpathway of ornithine degradation|unclassified**	47.5277366386
**PWY-5130: 2-oxobutanoate degradation I**	36.1826727153
**PWY-5130: 2-oxobutanoate degradation I|unclassified**	36.1826727153
**PWY-5384: sucrose degradation IV (sucrose phosphorylase)**	30.8495583198
**PWY-5384: sucrose degradation IV (sucrose phosphorylase)|unclassified**	30.8044156338
**PWY-621: sucrose degradation III (sucrose invertase)**	30.3355349627
**PWY-621: sucrose degradation III (sucrose invertase)|unclassified**	30.3142960675
**12DICHLORETHDEG-PWY: 1,2-dichloroethane degradation**	28.3462133221
**12DICHLORETHDEG-PWY: 1,2-dichloroethane degradation|unclassified**	28.3462133221
**GALACTUROCAT-PWY: D-galacturonate degradation I**	23.3112846169
**GALACTUROCAT-PWY: D-galacturonate degradation I|unclassified**	23.3112846169
**P161-PWY: acetylene degradation (anaerobic)**	18.2315319167
**P161-PWY: acetylene degradation (anaerobic)|unclassified**	18.2315319167
**PWY-4702: phytate degradation I**	17.7347835048
**PWY-4702: phytate degradation I|unclassified**	17.7347835048
**PWY-561: superpathway of glyoxylate cycle and fatty acid degradation**	15.0065800986
**PWY-561: superpathway of glyoxylate cycle and fatty acid degradation|unclassified**	14.9220064003
**PWY-5030: L-histidine degradation III**	13.0853575658
**PWY-5030: L-histidine degradation III|unclassified**	13.0799103853
**PWY-5417: catechol degradation III (ortho-cleavage pathway)**	9.8763843563
**PWY-5417: catechol degradation III (ortho-cleavage pathway)|unclassified**	8.3571360452
**PWY-5431: aromatic compounds degradation via β-ketoadipate**	9.8763843563
**PWY-5431: aromatic compounds degradation via β-ketoadipate|unclassified**	8.3571360452
**PWY-6185: 4-methylcatechol degradation (ortho cleavage)**	9.7727763710
**PWY-6185: 4-methylcatechol degradation (ortho cleavage)|unclassified**	9.7727763710

**PWY-6182: superpathway of salicylate degradation**	7.9983015761
**CATECHOL-ORTHO-CLEAVAGE-PWY: catechol degradation to β-ketoadipate**	6.9681151094
**CATECHOL-ORTHO-CLEAVAGE-PWY: catechol degradation to β-ketoadipate|unclassified**	5.8438278342

Table S10. Bins with genes associated with emerging contaminants used to generate Figure 6 bubble plot.

Bin	Taxonomy	Completeness	atzA	atzB	atzC	atzD	atzE	atzF	pheA	pheB	dmpD	xylF	catA	catB	catC	onpA	onpB	dntB	pcpB	pcpD
**33**	*Spirochaetia*	80	0	0	0	0	1	1	0	0	0	0	0	0	0	0	1	0	0	1
**35**	*Chlamydiales*	95,92	1	0	0	0	1	1	0	0	0	0	0	0	0	1	1	1	1	1
**41**	*Legionellales*	81,49	1	1	1	0	1	1	0	0	1	1	0	0	0	1	0	0	1	0
**58**	*Rickettsiales*	95,24	0	0	0	0	1	1	0	1	1	1	1	0	0	0	0	0	1	0
			Atrazine (Reductive pathway)										Phenytoin (oxidative pathways)							

bphA	hcaB	sul2	amdA	gdoA	tomF	ygiD
**0**	1	0	0	0	0	0
**0**	1	1	1	0	0	0
**0**	1	1	1	0	0	0
**0**	1	1	1	0	0	0
Carbamezapine oxidative pathways		Sulfamethoxazole Reductive pathways		Acetaminophen oxidative pathways		
